# Effects of Oral Butyrate on Blood Pressure in Patients With Hypertension: A Randomized, Placebo-Controlled Trial

**DOI:** 10.1161/HYPERTENSIONAHA.123.22437

**Published:** 2024-07-22

**Authors:** Barbara J.H. Verhaar, Madelief Wijdeveld, Koen Wortelboer, Elena Rampanelli, Johannes H.M. Levels, Didier Collard, Marianne Cammenga, Vanasa Nageswaran, Arash Haghikia, Ulf Landmesser, Xinmin S. Li, Joseph A. DiDonato, Stanley L. Hazen, Ingrid M. Garrelds, A.H. Jan Danser, Bert-Jan H. van den Born, Max Nieuwdorp, Majon Muller

**Affiliations:** Departments of Vascular Medicine (B.J.H.V., M.W., D.C., M.C., B.-J.H.v.d.B., M.N.), Amsterdam UMC location AMC, the Netherlands.; Experimental Vascular Medicine (K.W., E.R., J.H.M.L.), Amsterdam UMC location AMC, the Netherlands.; Department of Internal Medicine–Geriatrics, Amsterdam UMC location VUmc, the Netherlands (B.J.H.V., M.M.).; Amsterdam Cardiovascular Sciences, Diabetes and Metabolism, Atherosclerosis and Ischemic Syndromes, the Netherlands (B.J.H.V., M.W., K.W., E.R., B.-J.H.v.d.B., M.N., M.M.).; Amsterdam Gastroenterology Endocrinology Metabolism, Amsterdam UMC, University of Amsterdam, Netherlands (M.W., K.W., E.R.).; Amsterdam Institute for Infection and Immunity, Infectious Diseases, Cancer Immunology, the Netherlands (E.R.).; Department of Cardiology, Angiology and Intensive Care Medicine, Deutsches Herzzentrum der Charité, Campus Benjamin Franklin, Berlin, Germany (V.N., A.H., U.L.).; German Center for Cardiovascular Research (DZHK), Partner Site Berlin, Germany (A.H., U.L.).; Friede Springe-Cardiovascular Prevention Center at Charité, Charité-Universitätsmedizin Berlin Institute of Health, Germany (A.H., U.L.).; Department of Cardiovascular and Metabolic Sciences, Lerner Research Institute, Cleveland Clinic, OH (X.S.L., J.A.D.).; Department of Cardiovascular Medicine, Heart, Vascular and Thoracic Institute, Cleveland Clinic, OH (S.L.H.).; Department of Internal Medicine, Division of Pharmacology, Erasmus MC, Rotterdam, the Netherlands (I.M.G., A.H.J.D.).; Department of Public and Occupational Medicine, Amsterdam UMC, the Netherlands (B.-J.H.v.d.B.).

**Keywords:** blood pressure, butyric acid, feces, gastrointestinal microbiome, humans, hypertension

## Abstract

**BACKGROUND:**

The microbiota-derived short chain fatty acid butyrate has been shown to lower blood pressure (BP) in rodent studies. Nonetheless, the net effect of butyrate on hypertension in humans remains uncovered. In this study, for the first time, we aimed to determine the effect of oral butyrate on BP in patients with hypertension.

**METHODS:**

We performed a double-blind randomized placebo-controlled trial including 23 patients with hypertension. Antihypertensive medication was discontinued for the duration of the study with a washout period of 4 weeks before starting the intervention. Participants received daily oral capsules containing either sodium butyrate or placebo with an equivalent dosage of sodium chloride for 4 weeks. The primary outcome was daytime 24-hour systolic BP. Differences between groups over time were assessed using linear mixed models (group-by-time interaction).

**RESULTS:**

Study participants (59.0±3.7 years; 56.5% female) had an average baseline office systolic BP of 143.5±14.6 mm Hg and diastolic BP of 93.0±8.3 mm Hg. Daytime 24-hour systolic and diastolic BP significantly increased over the intervention period in the butyrate compared with the placebo group, with an increase of +9.63 (95% CI, 2.02–17.20) mm Hg in daytime 24-hour systolic BP and +5.08 (95% CI, 1.34–8.78) mm Hg in diastolic BP over 4 weeks. Butyrate levels significantly increased in plasma, but not in feces, upon butyrate intake, underscoring its absorption.

**CONCLUSIONS:**

Four-week treatment with oral butyrate increased daytime systolic and diastolic BP in subjects with hypertension. Our findings implicate that butyrate does not have beneficial effects on human hypertension, which warrants caution in future butyrate intervention studies.

**REGISTRATION:**

URL: https://onderzoekmetmensen.nl/; Unique identifier: NL8924.

**NOVELTY AND RELEVANCE**

**What Is New?**

- This randomized controlled clinical trial investigates the effect of oral butyrate supplementation in patients with hypertension. Despite many animal studies, there are no human studies investigating the direct effects of butyrate.

**What Is Relevant?**

- We found that butyrate increases daytime systolic and diastolic blood pressure. Although we explored potential mechanisms of action, we did not find a pathophysiological explanation for this finding in terms of renin-aldosterone suppression, sympathetic activation, or change in immunophenotype.

**Clinical/ Pathophysiological Implications?**

Our findings implicate that butyrate does not have beneficial effects on hypertension, warranting caution for its use as a nutritional supplement and its application in future intervention studies.

**See Editorial, pp 2137-2139**

Hypertension is the most important risk factor for preventable death worldwide and affects >1 billion adults.^1^ Uncontrolled hypertension impacts cardiovascular mortality through its secondary complications; 45% of mortality due to cardiac disease and 51% of mortality due to stroke can be attributed to hypertension.^2^ The pathogenesis of hypertension is assumed to be a combination of genetic predisposition and environmental factors, including dietary intake, sedentary behavior, smoking, and alcohol use. In addition, the gut microbiota could affect blood pressure (BP) regulation through its key metabolites, short chain fatty acids (SCFAs).^3^ SCFAs can be produced by gut microbes through fermentation of dietary fibers.^4^ Acetate (C2), propionate (C3) and butyrate (C4), the most abundant SCFAs, are produced in a 3:1:1 ratio.^5^ Most of the acetate and propionate is then absorbed, while the butyrate is predominantly used by colonocytes and absorbed in smaller quantities. Animal studies on butyrate have illustrated its BP-modulating effects but with reports disclosing both a BP-lowering or BP-increasing effect after butyrate administration.^6–9^ These incongruent findings could be explained by the antagonizing effects of different SCFA receptors on BP. Indeed, multiple receptors have been identified in a range of tissues including endothelium and vascular smooth muscle in the aorta and renal arteries.^10^ However, the exact interplay of SCFAs and their receptors in BP regulation remains incompletely understood.

Observational human studies have reported lower circulating SCFA levels and lower abundance of SCFA-producing bacteria in patients with hypertension.^11–14^ Consistent with these findings, a recently published intervention study with acetylated and butyrylated dietary fibers showed BP-lowering effects.^15^ However, the specific effects of administering individual SCFAs, such as butyrate alone, on BP and neurohormonal dynamics in humans remain unexplored. Despite the commercial availability of butyrate, there are currently no official recommendations for its use. Hence, in this randomized, placebo-controlled double-blind trial, we aimed to investigate the impact of oral butyrate treatment on BP in patients with hypertension.

## METHODS

### Data Availability

An extended Methods section is available in the Supplemental Methods. The clinical data that support the findings of this study are available from the corresponding author upon reasonable request. The raw 16S sequencing data are available in the European Nucleotide Archive, accession number PRJEB60224 (https://www.ebi.ac.uk/ena/browser/view/PRJEB60224).

### Participant Recruitment

Participants were recruited between March 2021 and July 2022 through online advertising. The study visits took place in Amsterdam UMC, Amsterdam, the Netherlands. All screened participants provided written informed consent, and all study procedures were approved by the Institutional Review Board of the Amsterdam UMC location AMC. The study was registered in the Dutch Trial Register (https://clinicaltrialregister.nl/nl/trial/22936; unique identifier: NL8924). We included Dutch participants aged 40 to 65 years with hypertension (office systolic BP between 140 and 160 mm Hg or diastolic BP between 90 and 100 mm Hg) without treatment or the use of 1 BP-lowering drug. The BP range aligns with grade I hypertension in the 2018 European Society of Cardiology/European Society of Hypertension hypertension guidelines, whereas in the 2017 American College of Cardiology/American Heart Association guidelines, these values would classify as grade II hypertension.^16,17^ Participants were required to have a body mass index below 27 kg/m^2^. Postmenopausal women were eligible for the study, whereas premenopausal women were not included due to potential hormonal influences on both BP and gut microbiota composition.^18,19^ Exclusion criteria were the use of β-blockers, current smoking, known secondary causes of hypertension, a history of cardiovascular disease, impaired renal function, diabetes, or severe gastrointestinal disease. Patients on BP-lowering drugs were asked to discontinue this medication for the duration of the study, with a 4-week washout period monitored by weekly home BP measurements before the baseline visit. We incorporated a safety criterion in our protocol, dictating that participants with >180 systolic or >110 diastolic office BP would be excluded from further participation in the trial; none of the patients in this trial were excluded during the study for safety reasons.

The primary outcome was defined in the study protocol as daytime 24-hour systolic BP. Before the study, we performed a power calculation resulting in a target of 50 participants. This sample size was unfortunately not reached due to the impact of the SARS-CoV-2 pandemic, and the study was terminated prematurely at 23 inclusions. We used the Consolidated Standards of Reporting Trials (CONSORT) checklist in writing our report.^20^

### Randomization, Blinding, and Intervention

Included participants were randomized using a stratified alternating block randomization (1:1 ratio; block sizes 4, 6, and 8), with 2 strata for age (≤50 and >50 years) and sex. Physicians and study participants were blinded to treatment allocation, until the statistical analyses were completed in a blinded fashion. Participants in the butyrate treatment arm received 3.9 g of sodium butyrate daily for 4 weeks, divided into 13 capsules taken in the morning and evening (total of 26 capsules per day). Placebo arm participants received an equal number of identical placebo capsules containing an equivalent amount of sodium chloride (2.0 g). Both groups had an additional daily sodium load of 798 mg. Compliance was monitored by asking about missed doses at 2 and 4 weeks and by counting returned capsules. Participants reported adverse effects, with only those deemed possibly related to the investigational product reported.

Data and samples were collected around 4 study visits that took place in the morning after an overnight fast. We instructed participants not to make any impactful changes to their diet during their study participation. Before the baseline visit, participants were asked to keep a nutritional diary for 3 days and to collect fecal samples and 24-hour urine. During the visit, BP data were collected, multifrequency body impedance analysis was performed, and venous blood samples were drawn. Patients were connected to an ambulatory 24-hour measurement device that measured until the next morning. After completing the 24-hour BP measurement, they were instructed to take the first capsules. The second, midterm visit took place after 2 weeks of treatment. This was a shorter visit that included a compliance and adverse events check, office BP measurement, weighing, and body impedance analysis measurement. All measurements were repeated during the third visit, at the end of the intervention period, after ≈4 weeks. The fourth and last visit took place 1 week after discontinuation of the treatment to assess whether the treatment effects would persist longer than the treatment duration.

### BP Measurements

Office BP was measured by a semiautomatic oscillometric device (Microlife WatchBP Home; Microlife AG, Switzerland) following the European Society of Hypertension guidelines.^17^ Three BP measurements were taken in seated position after 5 minutes of rest, and the average BP was calculated from the second and third measurements. During the study visit, a 5-minute continuous BP recording was made with a Nexfin device (Nexfin; Edwards Lifesciences, Irvine, CA). We exported raw beat-to-beat data of interbeat intervals, systolic BP, and dP/dt and performed analyses in Matlab (R2019a; MathWorks, Inc) to calculate heart rate variability (HRV) and cross-correlation baroreceptor sensitivity.^21^ Twenty-four-hour ambulatory BP measurements were performed with oscillometric Spacelabs 90227 devices (Spacelabs Healthcare, Issaquah, WA). The devices were programmed to measure 4× per hour during the day (7:00 am to 10:00 pm) and 2× per hour during the night (10:00 pm to 7:00 am). A valid 24-hour recording was defined as a recording with >70% of the programmed measurements being successful; all recordings in this study were valid. Participants reported their bedtime and waking time to enable the calculation of average daytime and nighttime BP.

### Sample Collection, Biochemistry, and 16S Sequencing

Blood samples were collected in heparin, EDTA, or serum gel BD Vacutainer tubes and kept on ice until further processing. Plasma and serum that were not directly used for measurements were stored at −80 °C. Serum concentrations of IL-6 (interleukin-6) and IFNγ (interferon-γ) were determined with the high-sensitivity human ELISA kits (Invitrogen, Thermo Fisher Scientific) according to the manufacturer’s procedure. Plasma serotonin concentrations were determined in EDTA plasma samples using an high-performance liquid chromatography method, while for plasma SCFA analysis, gas chromatography–mass spectrometry was used. Renin concentrations were measured in EDTA plasma with an immunoradiometric assay (Beckman Coulter, Immunotech, Prague, Czech Republic) with an active site-directed radiolabeled antibody with high specificity for renin (as opposed to prorenin) and a lower detection limit of 2 pg/mL. Plasma aldosterone levels were measured in EDTA plasma with a solid-phase radioimmunoassay (Demidetec Diagnostics, Kiel, Germany) with a lower detection limit of 12 pg/mL. Fractional excretion of sodium was calculated from 24-hour urine and plasma sodium and creatinine.^22^ Fecal samples were collected by the participants the day before the study visit and were stored overnight in a freezer. Frozen samples were transported to the hospital in a provided cooling bag and stored at −80 °C. After study completion, fecal calprotectin and SCFA levels were measured.^23,24^ The fecal SCFA levels were corrected for the dry weight of the samples. To determine gut microbiota composition, 16S rRNA sequencing of samples was performed.^25,26^ After bioinformatic processing and rarefaction to 14 000 counts per sample, the gut microbiota data consisted of 62 samples and 2405 amplicon sequence variants (ASVs).^27^

### Monocyte Isolation From Peripheral Blood and Ex Vivo Stimulations

Peripheral blood mononuclear cells (PBMCs) were isolated from blood with a density gradient centrifugation protocol. Monocytes were isolated using the Pan Monocyte Isolation Kit and MACS MS columns (Miltenyi Biotec, Bergisch Gladbach, Germany) according to the manufacturer’s procedure for the simultaneous isolation of untouched monocyte populations, including classical (cluster of differentiation [CD]14^+^CD16^−^), nonclassical (CD14^dim^CD16^+^), and intermediate (CD14^+^CD16^+^) monocytes. To determine the proportions of monocyte subsets, monocytes were stained with CD11b, CD14, and CD16 antibodies and analyzed on a BD LSRFortessa Cell Analyzer. Data were analyzed using the FlowJo (v.10) software. To assess the monocyte inflammatory profile, monocytes were seeded in 96-well plates and stimulated with either control medium (200 µL of 5% heat-inactivated fetal bovine serum-supplemented Roswell Park Memorial Institute [HI-FBS-RPMI] 1640 medium with GlutaMAX and penicillin-streptomycin), 100 µmol/L palmitate, or 1 ng/mL lipopolysaccharide for 24 hours in a 37 °C 5% CO_2_ incubator. The supernatant was harvested and stored at −80 °C; adherent monocytes were washed, and the medium was replaced. After 5 days, the monocytes were restimulated with lipopolysaccharide 10 ng/mL for 24 hours, to induce trained immunity in the palmitate-trained monocytes and immunotolerance in the lipopolysaccharide-trained monocytes. Concentrations of IL-6 and TNFα (tumor necrosis factor-α) were measured in the collected monocyte supernatant (in quadruplicate) using the OptEIA human TNFα and IL-6 ELISA sets (BD Biosciences). Cytokine concentrations from the first stimulation are shown as the difference between stimulated and control conditions, normalized for total monocyte count, while data from the second stimulation are shown as fold changes compared with the first stimulation.

### T-Cell Phenotyping by Flow Cytometry

Changes in T-cell subsets upon placebo or butyrate treatment were investigated using a 12-color panel designed to identify all major CD4 T-cell populations. One vial of frozen PBMC was thawed, and 1 million PBMCs were used for the staining. PBMCs were first stained with a cocktail of fluorochrome-labeled antibodies (BV421-conjugated anti-CD194/CCR4 [C-C chemokine receptor 4], BB700-conjugated anti-CD185/CXCR5 [C-X-C chemokine receptor 5], BV786-conjugated anti-CD196/CCR6, PE [phycoerythrin]-conjugated anti-CD294/CRTH2 [prostaglandin D2 receptor 2], PE-Cy7 [cyanine-7]-conjugated anti-CD183/CXCR3; BD Biosciences) for 10 minutes at 37 °C in the dark; afterward a new cocktail of fluorochrome-labeled antibodies (brilliant violet [BV]605-conjugated anti-CD45RA, BV711-conjugated anti-CD127, brilliant blue [BB]515-conjugated anti-CD25, APC [allophycocyanin]-conjugated anti-CCR10, Alexa Fluor 700-conjugated anti-CD3, APC-H7-conjugated anti-CD4, PE-Cy5-conjugated anti-CD8; BD Biosciences) was added to prestained cells for additional 20 minutes at room temperature in the dark. Stained PBMCs were analyzed using a 4-laser FACSymphony A1 Cell Analyzer. The obtained flow cytometric data were analyzed using the FlowJo (v.10) software. Gating strategies can be found in Figure S14.

### Statistics

All statistical analyses were performed in RStudio (v.2023.9.1.494) using R (v.4.2.1). All scripts were made publicly available in a Github repository (https://github.com/barbarahelena/beam-study). Treatment effects of oral butyrate on all primary and secondary outcomes were assessed using linear mixed models (lme4 [v1.1.31] and afex [v.1.2.0] packages). Missing data in these analyses were limited to 1 missing continuous BP recording at the baseline time point. This subject was, therefore, excluded from the cross-correlated baroreceptor sensitivity (xBRS) and HRV analyses. In the unadjusted linear mixed models, participant number was included as a random effect, and the treatment group, time, and the interaction term group×time were included as fixed effects. The effect of treatment was considered significant if the group×time estimate was significant (*P*<0.05). In the adjusted model for ambulatory and office BP, the covariates age, sex, body mass index, sodium intake (nutritional diary), and compliance (number of capsules left) were included. In the model for plasma SCFA levels, we included compliance and the time of the study visit (as proxy for time from morning dose to sample collection) as covariates. In the model for fecal SCFA levels, we only included compliance as a covariate because samples were already collected before the study visit. A detailed explanation on the gut microbiota composition analyses can be found in the Supplemental Methods.

## RESULTS

### Population Characteristics

We included 23 participants, of whom 11 subjects were randomly assigned to the placebo group and 12 subjects to the butyrate group (Table; Figure S1). Twelve participants (52.2%) used BP-lowering drugs and had to go through a washout period of 4 weeks before the start of the intervention. Two of these participants (1 in the butyrate group and 1 in the placebo group) dropped out before the baseline visit because of complaints of headaches. At baseline, that is, after the washout period, office systolic BP was on average 143.5±14.6 mm Hg and diastolic BP, 93.0±8.3 mm Hg. Dietary patterns including total energy intake, fiber intake, and alcohol consumption did not change significantly or differentially between the treatment groups over the intervention period (Figure S2). Compliance to the intervention (percentage of used capsules) was estimated from the number of capsules that participants returned after study completion and was on average 98.3±2.7% for the butyrate group and 96.6±3.6% for the placebo group. Four adverse events were recorded during the study intervention in 3 participants allocated to the placebo group and included complaints of constipation and mild headaches.

**Table.**
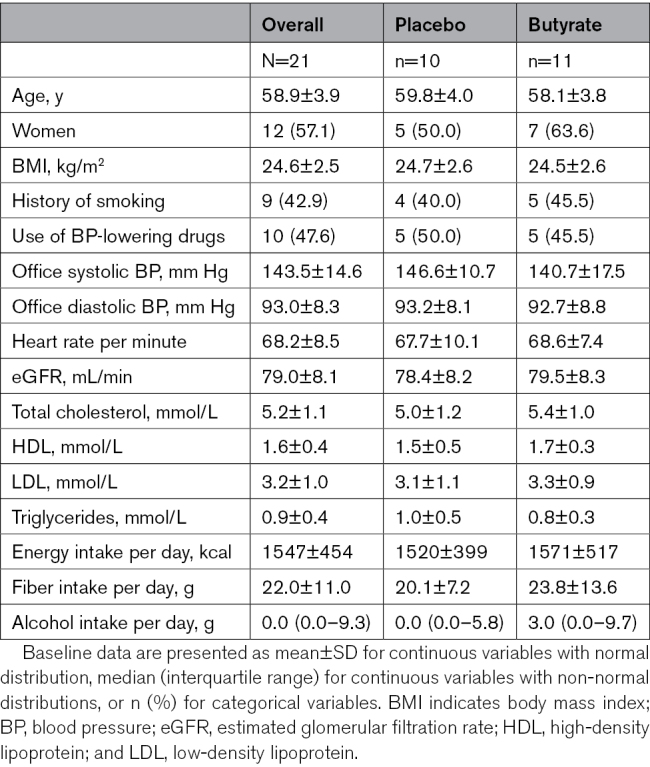
Population Characteristics

### Butyrate Increases Daytime Systolic and Diastolic BP Compared With Placebo

**Figure 1.**
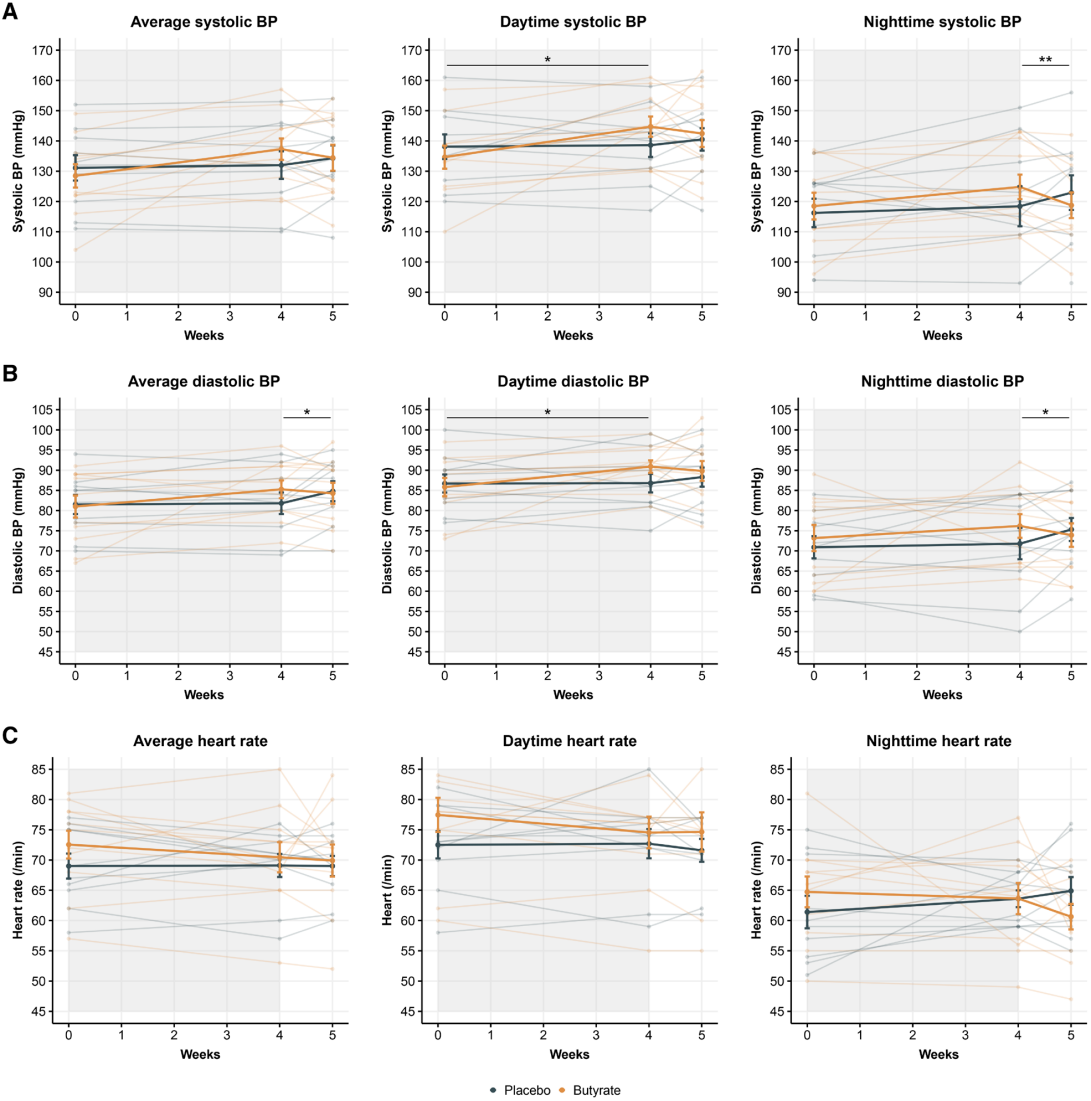
**Ambulatory blood pressure (BP) changes. A**, Systolic blood pressure (overall, daytime and nighttime). **B**, Diastolic blood pressure (overall, daytime and nighttime). **C**, Heart rate (overall, daytime and nighttime). Ambulatory BP data at baseline, at the end of the intervention (week 4), and after the intervention (week 5). The darker lines represent the group mean over time, with error bars for SEs, while the lighter lines indicate the individual subjects’ BP over time. Group differences over time (group×time) were tested with linear mixed models between baseline and end of intervention (weeks 0–4), and end of intervention and follow-up after intervention (weeks 4–5). *P<0.05.

Daytime 24-hour systolic and diastolic BP significantly increased over the intervention period in the butyrate compared with the placebo group (Figure 1). While the daytime systolic BP remained essentially unchanged in the placebo group (138.1±12.9 mm Hg at baseline to 138.6±12.3 mm Hg at 4 weeks), it increased from 134.6±12.6 to 144.7±11.1 mm Hg in the butyrate group during the intervention. One week after stopping the intervention, the effect of butyrate treatment disappeared and the difference between the 2 treatment arms converged. While nighttime systolic and diastolic BP increased more over time in the butyrate group, the group differences were not statistically significant. However, the decrease in both systolic and diastolic nighttime BP after discontinuing the treatment was significantly steeper for the butyrate group. We also performed adjusted linear mixed models (Figure S3), showing that over the 4-week treatment, there was a 9.63-mm Hg increase ([95% CI, 2.02–17.20] *P*=0.02) in daytime 24-hour systolic BP and 5.08-mm Hg increase ([95% CI, 1.34–8.78] *P*=0.02) in diastolic BP in the butyrate compared with the placebo group. The total (*P*=0.06) and nighttime BP showed similar trends albeit not statistically significant. In addition, 24-hour heart rate data showed a small and nonsignificant decrease in the butyrate group. Office BP showed a similar trend as 24-hour BP with a slight increase in systolic and diastolic BP in the butyrate group, but these changes were not significant compared with placebo (Figure S4).

### Butyrate Has No Differential Effects on Neurohormonal Responses and Sodium Balance

**Figure 2.**
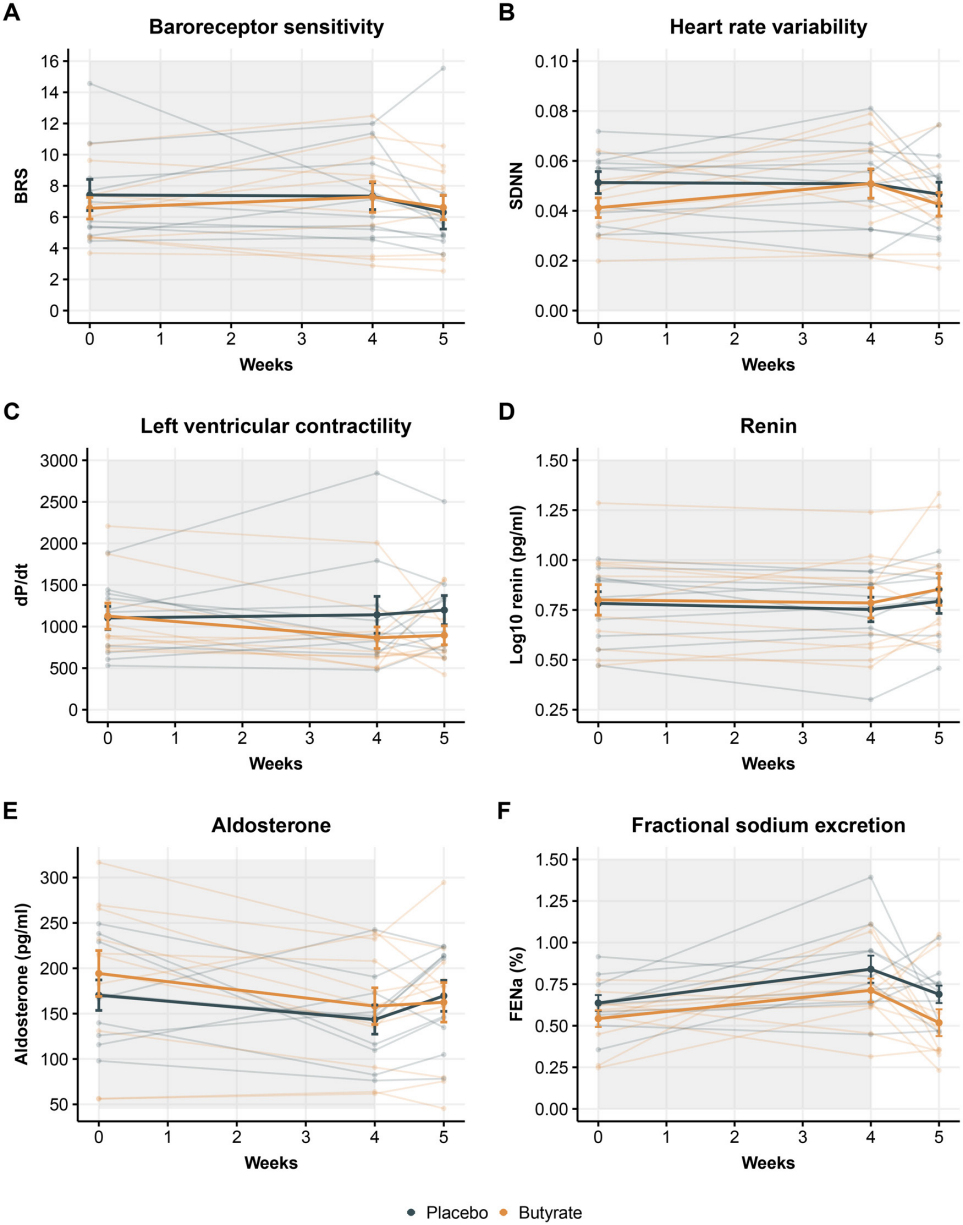
**Effects on neurohormonal responses and sodium balance. A**, Baroreceptor sensitvity. **B**, Heart rate variability. **C**, Left ventricular contractility. **D**, Renin levels. **E**, Aldosterone levels. **F**, Fractional sodium excretion. Neurohumoral response and sodium balance at baseline, at the end of the intervention (week 4), and after the intervention (week 5). BRS indicates baroreceptor sensitivity; FENa, fractional excretion of sodium; and SDNN, standard deviation of N-N intervals.

xBRS and HRV as indicators of sympathetic activation showed no significant group differences, although we observed a trend toward an increased HRV in the butyrate group (group×time: *P*=0.078; Figure 2A and 2B). dP/dt as an indicator of left ventricular contractility decreased during the intervention in the butyrate group, but the difference with the placebo group was not significant (Figure 2C; group×time: *P*=0.122). Plasma renin concentrations remained stable, while aldosterone levels decreased in both groups in line with the increased sodium load during treatment (Figure 2D and 2E). Fractional excretion of sodium increased significantly during the intervention in both groups (both groups, *P*<0.05) and was, therefore, not significantly different between groups (Figure 2F). Using body impedance analyses, we were able to monitor changes in total body water during the study period including the proportion of extracellular body water, which did not change significantly between the groups (Figure S5).

### Butyrate Administration Increases Plasma Butyrate Levels

**Figure 3.**
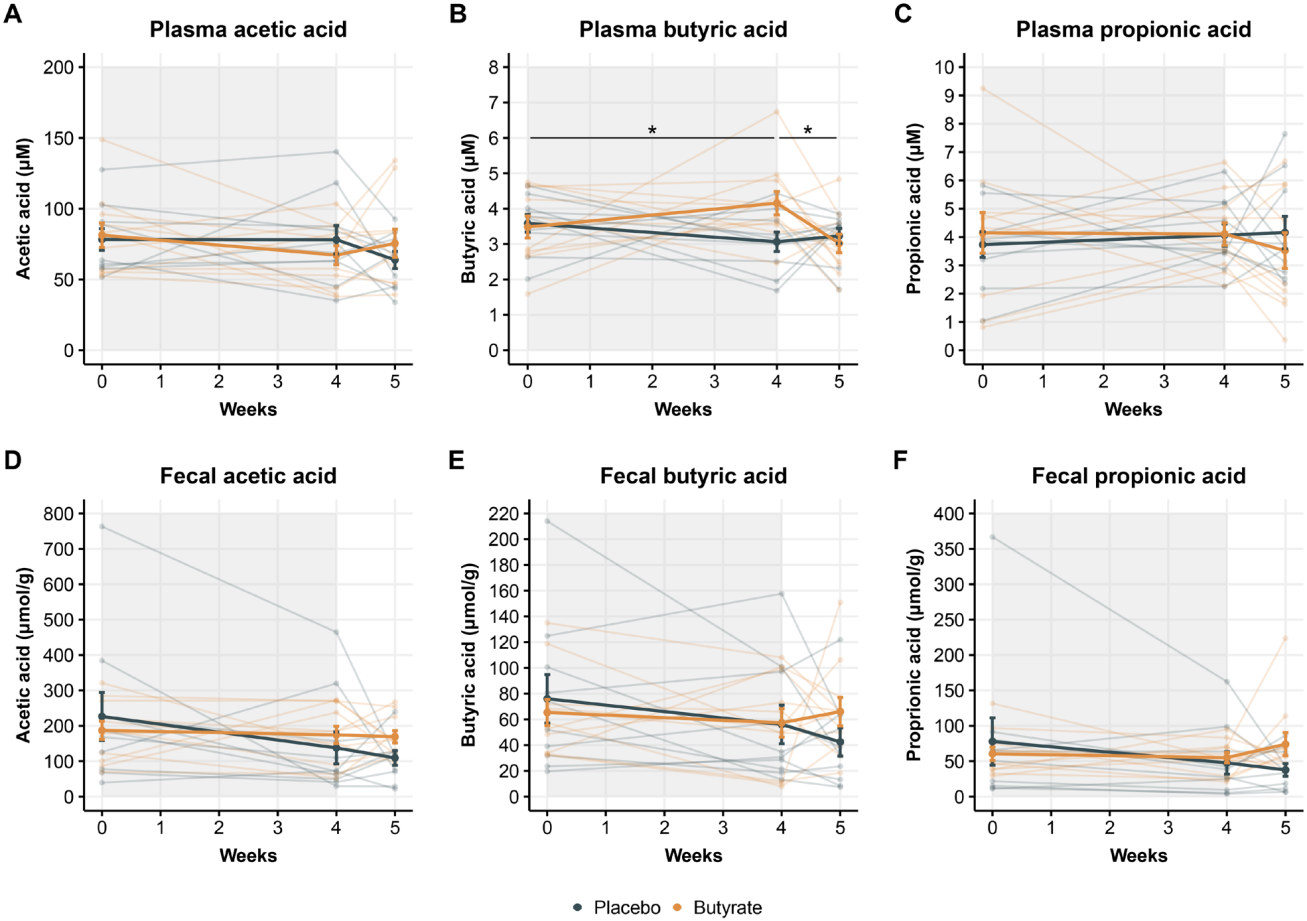
**Changes in plasma and fecal short chain fatty acid (SCFA). A**, Baroreceptor sensitvity. **B**, Heart rate variability. **C**, Left ventricular contractility. **D**, Renin levels. **E**, Aldosterone levels. **F**, Fractional sodium excretion. Plasma and fecal SCFA concentrations at baseline, at the end of the intervention (week 4), and after the intervention (week 5). Fecal SCFA levels are shown in μmol/g dry weight; plasma SCFAs are shown in μmol/L.

Of the plasma SCFA, only butyrate levels changed between groups during the intervention (group×time: *P*=0.047), while other SCFAs showed no group differences (Figure 3A through 3C). Mean butyrate levels increased from 3.48±1.04 to 4.16±1.10 μmol/L, while in the placebo group, levels decreased from 3.58±0.79 to 3.06±0.86 μmol/L. After the intervention, butyrate levels of the oral butyrate group normalized, while the levels in the placebo group remained stable (group×time: *P*=0.015). In contrast, fecal SCFA concentration changes were not significantly different between treatment groups (Figure 3D through 3F). We additionally measured serotonin levels and found no group differences over time (Figure S6).

Gut microbiota composition as determined with 16S sequencing showed no clear shifts in composition during treatment (Figure S7A). In line, β-diversity was not significantly different between the visits in each treatment group (Figure S7B; permutational analysis of variance: *P*>0.05). α-Diversity increased slightly in the butyrate group during the intervention and was slightly higher than the placebo group during treatment (Figure S7C and S7D; Faith phylogenetic diversity: *P*=0.019). At the ASV level, linear mixed models showed no significant changes between baseline and the end of the intervention (group×time interaction) after multiple testing correction (Figure S8).

### Butyrate Does Not Affect Systemic Inflammation, Monocyte Inflammatory Responses, or Monocyte and T-Cell Profiles

**Figure 4.**
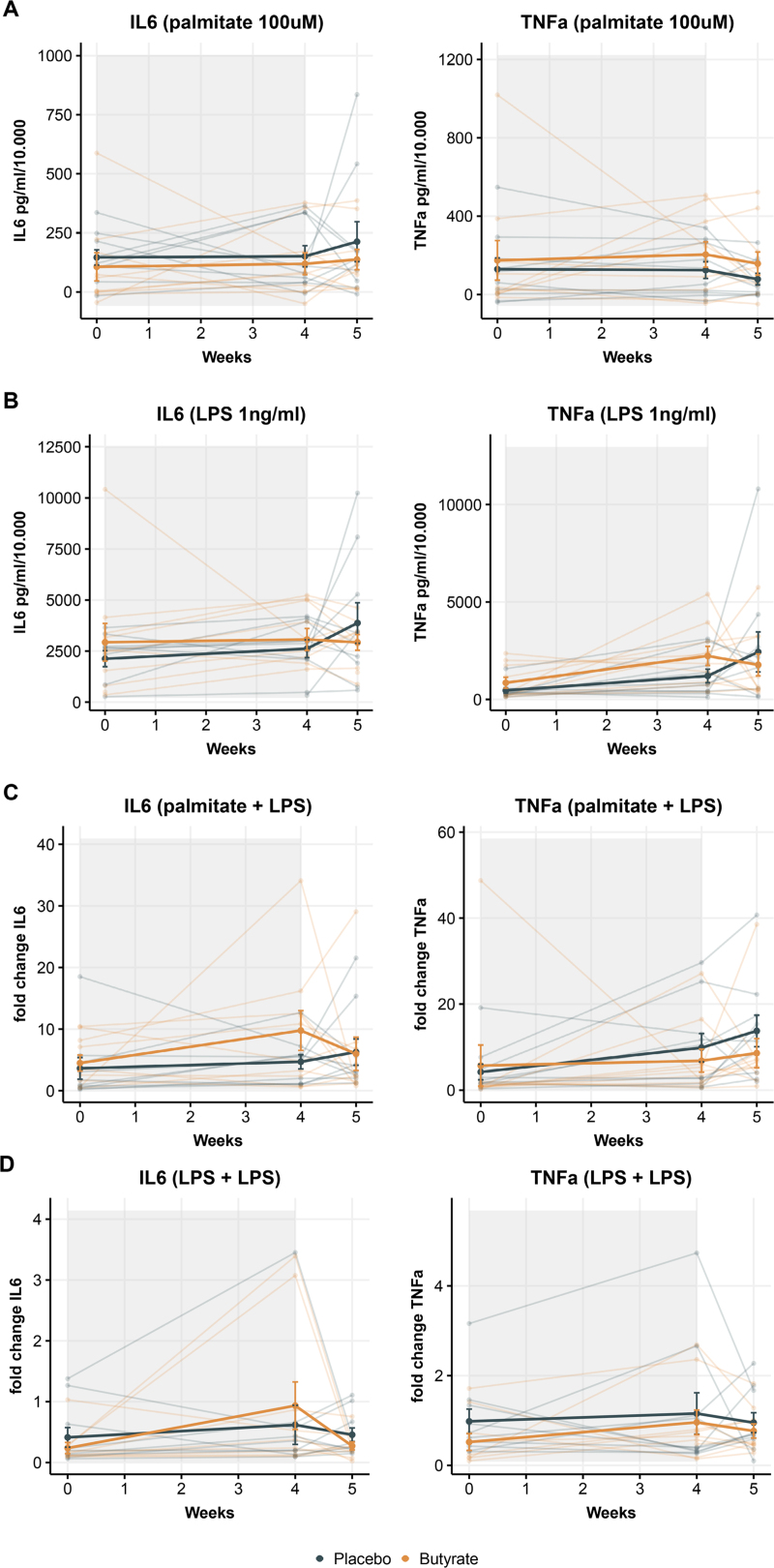
**Cytokine levels after monocyte stimulations.** IL-6 (interleukin-6) and TNFα (tumor necrosis factor-α) concentrations in supernatant of monocytes. **A**, Palmitate-stimulated monocytes. **B**, Lipopolysaccharide (LPS)-stimulated monocytes. **C**, Trained immunity model: LPS stimulation of palmitate-trained monocytes. **D**, Immunotolerance model: LPS stimulation of LPS-trained monocytes.

Since butyrate has been shown by numerous reports to act as an anti-inflammatory agent via the inhibition of NF-κB (nuclear factor-κB) activation,^28–30^ we questioned whether oral butyrate supplementation would inhibit the inflammatory responses of circulating monocytes exposed to palmitate or lipopolysaccharide. Monocytes isolated at baseline, after treatment (week 4), and after stopping therapy (week 5) were stimulated with palmitate or lipopolysaccharide for 24 hours to induce IL-6 and TNFα production. Neither sodium chloride treatment nor sodium butyrate supplementation influenced the inflammatory responses of monocytes upon TLR4 (toll-like receptor 4)-signaling activation (Figure 4A and 4B). As hypertensive mechanical stretch of the endothelial layer has been shown to alter the monocyte activity and phenotype, we investigated any potential effect of butyrate on the proportions of classical (CD14^+^CD16^−^), intermediate (CD14^+^CD16^+^), and nonclassical (CD14^dim^CD16^+^) monocyte subpopulations, which showed no group differences (Figure S9). To investigate whether the reported inhibitive effect of butyrate on trained immunity would also occur in the context of hypertension,^31^ stimulated monocytes were restimulated with 1 ng/mL lipopolysaccharide to induce trained immunity after palmitate priming or immunotolerance after lipopolysaccharide priming. In the trained immunity model, with restimulation of palmitate-trained monocytes, the butyrate group showed a small nonsignificant increase in IL-6 production after treatment with no change in TNFα levels (Figure 4C). Similarly, in the immunotolerance model, lipopolysaccharide-trained monocytes restimulated with lipopolysaccharide displayed similar trends in IL-6 and TNFα responses between the treatment groups (Figure 4D). In addition, we measured circulating cytokines in serum, which showed a nonsignificant increase in circulating IFNγ in the butyrate compared with placebo group and no change in IL-6 levels (Figure S10). In light of this observation and the notion that IFNγ is a typical T cell–derived proinflammatory cytokine, we also extensively analyzed the T-cell immunoprofile. To this end, we used a 12-color antibody panel designed for the resolution of CD8 T cells and CD4 T-cell subsets, including effector helper T (Th) cells Th1, Th2, Th9, Th17, Th22, ThG (T-helper G), follicular helper, and regulatory T cells. No significant changes were observed in the proportions of total CD8^+^/CD4^+^ T cells or in the specific subset of CD4^+^ memory T cells, except for an unexpected increase in Th9 CD4^+^ T cells in the butyrate compared with the placebo group during the treatment period (Figures S11 and S12). Lastly, as a general marker of intestinal inflammation, we measured the levels of fecal calprotectin, which were found to be similar between the groups over time (Figure S13).

## DISCUSSION

In this randomized placebo-controlled trial, we found that oral sodium butyrate supplementation in patients with mild hypertension increased daytime systolic and diastolic BP compared with sodium chloride placebo. Oral butyrate increased plasma levels of butyrate yet no other SCFA; therefore, these effects are likely direct effects of butyrate. We did not find effects on renin and aldosterone levels, sympathetic activation, body composition, major immunophenotype, or circulating cytokines. While the exact mechanism of the BP-increasing effect of butyrate supplementation remains to be elucidated, this study shows that butyrate does not have antihypertensive effects.

The observed increase in daytime systolic BP in our study is somewhat unexpected considering the evidence from animal studies, which predominantly show BP-lowering effects.^8,13,32–34^ A recent randomized controlled trial with acetylated and butyrylated high amylose maize (HAMSAB), which ensures a sustained colonic delivery of acetate and butyrate at high concentrations, reported a reduction in ambulatory BP after 3 weeks of treatment.^15^ However, it is challenging to isolate the effects of SCFA from those of fiber intake on microbiota structure and metabolic capacity. For instance, fiber intake increases endogenous production of acetate, butyrate, and propionate and might additionally alter the ratio of plasma SCFA (acetate:butyrate:propionate). In contrast, other preclinical and observational studies pointed to potential BP-increasing effects of butyrate. Two animal studies showed the potential of butyrate to exacerbate hypertension and to induce vascular calcification,^7,35^ in addition to 2 human studies that reported positive associations of plasma butyrate with BP and arterial stiffness.^11,36^

We explored several mechanisms underlying the BP-increasing effects of butyrate based on evidence from animal studies. We measured renin and aldosterone levels, considering the role of the SCFA receptor Olfr78 (olfactory receptor 78; human orthologue OR51E2), known to mediate renin release in rodents.^6,34,37^ We did not find any group differences between butyrate and placebo. However, it is possible that small effects of butyrate on the renin-aldosterone system were obscured, as the increased sodium load in both treatment arms may have already suppressed the system, as illustrated by the increase in fractional sodium excretion and decrease in aldosterone levels. The trend toward lower heart rates in the butyrate group could point to sodium retention. In addition, butyrate could affect the intrarenal renin-angiotensin system, which may not be reflected in systemic levels.^34^ We also explored the effects of butyrate on the sympathetic nervous system, since SCFA receptors such as FFAR3 (free fatty acid receptor 3) are expressed in the hypothalamic paraventricular nucleus and butyrate is able to cross the blood-brain barrier.^38^ We did not find effects of oral butyrate on xBRS and HRV as proxies of sympathetic balance. Butyrate might also exert direct vasoactive effects, given the expression of SCFA receptors such as FFAR2 (free fatty acid receptor 2) and FFAR3 in the vasculature.^33,35^ However, within this study, it was not possible to directly assess the expression of these receptors in the relevant human tissues.

Although butyrate is widely recognized as a potent anti-inflammatory mediator,^39–41^ our immunologic analyses aimed to uncover butyrate-mediated effects on monocytes, and trained immunity did not confirm these effects in the context of hypertension. Additionally, we did not observe any changes in inflammatory markers, such as serum IL-6 and IFNγ, or in fecal calprotectin levels. These results align with previous studies, including the HAMSAB trial and a recent oral butyrate trial in patients with inflammatory bowel disease, which also did not report significant changes in several of these markers.^42^ Besides monocytes, T lymphocytes are activated in hypertensive conditions and support the pathogenesis of hypertension through cytokine secretion and an adverse impact on vascular remodeling and renal natriuretic responses.^43–49^ Classical changes within the T-cell compartment associated with hypertension include the expansion of Th1 and Th17 subsets, accompanied by augmented IFN-γ and IL-17 levels, and decreased in regulatory T cells.^50,51^ In our immunophenotyping analysis, we did not find any treatment-induced changes in these relevant T-cell populations; instead, the only significant difference was in the increased frequency of Th9 cells after 4 weeks of butyrate supplementation. Th9 cells are one of the most recently identified subsets of CD4^+^ T helper cells. They are characterized by the secretion of IL-9, as well as IL-10 and IL-21, and are described to play a proinflammatory role in allergic reactions; however, they also exert protection against parasitic helminth infections and are endowed with robust anticancer capacity.^52^ Yet, the link between Th9 and hypertension remains undiscovered.^46^ Similarly, this is the first report disclosing an induction of Th9 upon butyrate intake. Our findings are in contrast to a previous murine study, in which butyrate appeared to suppress Th9 differentiation in a mouse model of lung inflammation.^53^ Nonetheless, differences in species and disease context may underlie these discordant findings.

Most evidence to date has pointed to a beneficial role of SCFA, butyrate in particular, in diverse cardiometabolic and intestinal conditions.^54,55^ In contrast, our study suggests that butyrate may elevate BP and hence potentially negatively impact cardiovascular health. An increase of almost 10 mm Hg in systolic BP or of 5 mm Hg in diastolic BP would translate to substantial higher risk of cardiovascular mortality.^56^ Given that sodium butyrate is readily available commercially and already used as a nutritional supplement, our findings underscore the need for caution in its use by patients with hypertension. Future studies should investigate the underlying mechanisms explaining our findings, particularly focusing on sodium retention and the renin-aldosterone system and preferably conducted without a concomitant sodium load, for instance, through the use of tributyrine.

Our study has several limitations. We terminated the trial prematurely due to the SARS-CoV-2 pandemic, resulting in a smaller sample size that limits our ability to detect subtle changes explaining our findings. Although our primary outcome, daytime systolic BP, significantly increased, the trend in nighttime BP did not reach significance, possibly due to the shorter duration and lower frequency of nighttime measurements compared with daytime. While we observed a rise in plasma butyrate levels within the butyrate group, it is important to acknowledge that the increase, although statistically significant, was relatively modest, which can likely be attributed to the short half-life of butyrate,^57^ coupled with the variability introduced by the time between the morning dosage and the blood draw of the study visit. Future studies could consider to measure plasma, urine, and fecal levels at multiple time points to get more insight into the pharmacokinetics of oral sodium butyrate. In addition, the administered capsules release butyrate in the small intestine and not in the colon where absorption occurs physiologically. Our intervention might, therefore, not reflect the local effects of colonic butyrate production, although in this study, we were primarily interested in the systemic effects of oral butyrate on BP.

Strengths of this study include the 24-hour ambulatory BP as the primary outcome, which is more accurate than office BP measurements, and the broad phenotyping of participants, which enabled us to assess the potential mechanisms of butyrate. In addition, we planned an extra (washout) study visit 1 week after stopping the intervention, to assess whether there were any prolonged effects, which showed that the effects of butyrate disappear rapidly after discontinuation. To our knowledge, this is the first trial to investigate the direct effect of butyrate on BP in a placebo-controlled trial with patients with hypertension.

In conclusion, we found that oral butyrate supplementation has a BP-increasing effect in patients with mild hypertension, suggesting that the BP-modulating pathways of SCFAs are more complex than previously thought. Our findings implicate that butyrate does not have beneficial effects on hypertension, which warrants caution for its use as nutritional supplement and as application in future intervention studies.

## PERSPECTIVES

Despite advances in therapy, BP control is not reached in all patients, urging the development of novel strategies to alleviate the burden of hypertension and associated end-organ failures. Our study investigated the effects of butyrate on BP in humans. We show that butyrate increases daytime systolic BP, which warrants caution for the use sodium butyrate as nutritional supplement by patients with hypertension. Although we did not find a clear mechanistic explanation, we speculate that sodium retention and subtle effects on the renin-aldosterone system could potentially play a role.

## ARTICLE INFORMATION

### Acknowledgments

The authors thank Nagina Simkhada, BSc, for her assistance in the medication preparation and Iris Visser, MD, for her help in the recruitment of participants.

### Sources of Funding

B.J.H. Verhaar is appointed on an Amsterdam Cardiovascular Sciences grant (ACSPhD2019P003) and a TransAtlantic Networks of Excellence Program grant (33.17CVD01) from Fondation Leducq. K. Wortelboer is appointed on a Novo Nordisk Foundation CAMIT grant 2018 (28232) to Max Nieuwdorp. E. Rampanelli is supported by a ZONMW VIDI grant 2023 (09150172210050) and AUMC Starter Grant. M. Nieuwdorp is supported by a personal ZonMw-VICI grant 2020 (09150182010020). S.L. Hazen also reports support by grants from the NIH and Office of Dietary Supplements (P01 HL147823, R01 HL103866). A. Haghikia is a participant in the BIH Charité Advanced Clinician Scientist Pilot Program funded by the Charité–Universitätsmedizin Berlin and the Berlin Institute of Health.

### Disclosures

M. Nieuwdorp is on the scientific board at Caelus Pharmaceuticals, the Netherlands. S.L. Hazen reports being named as a coinventor on pending and issued patents held by the Cleveland Clinic relating to cardiovascular diagnostics and therapeutics, being a paid consultant formerly for Procter & Gamble, and currently being with Zehna Therapeutics. He also reports having received research funds from Procter & Gamble, Zehna Therapeutics, and Roche Diagnostics and being eligible to receive royalty payments for inventions or discoveries related to cardiovascular diagnostics or therapeutics from Cleveland HeartLab, a wholly owned subsidiary of Quest Diagnostics, Procter & Gamble, and Zehna Therapeutics. The other authors report no conflicts.

### Supplemental Material

Supplemental Methods

Figures S1–S14

Major Resources Table

References 16, 17, 19, 21, 22, 23

## Supplementary Material


