# Supplementary material for: Effects of Oral Butyrate on Blood Pressure in Patients With Hypertension: A Randomized, Placebo-Controlled Trial
Source: Hypertension. 2024 Jul 22;81(10):2124–36. doi: 10.1161/HYPERTENSIONAHA.123.22437 (PMC11404767; doi:10.1161/HYPERTENSIONAHA.123.22437)
Supplement: Supplementary file 1 [file hyp-81-2124-s001.pdf]

ONLINE SUPPLEMENTS

Effects of oral butyrate on blood pressure in patients with hypertension: a randomized, placebo-controlled trial

Barbara J.H. Verhaar,[1,2,3*] Madelief Wijdeveld,[1,3,5] Koen Wortelboer,[3,4,5] Elena Rampanelli,[3,4,5,6] Johannes H.M. Levels,[4] Didier Collard,[1] Marianne Cammenga,[1] Vanasa Nageswaran,[7] Arash Haghikia,[7,8,9] Ulf Landmesser,[7,8,9] Xinmin S. Li,[10] Joseph A. DiDonato,[10] Stanley L. Hazen,[10,11] Ingrid M. Garrelds,[12] A.H. Jan Danser,[12] Bert-Jan H. van den Born,[1,3,13] Max Nieuwdorp,[1,3] Majon Muller[2,3]

1 Department of Vascular Medicine, Amsterdam UMC, location AMC, Amsterdam, The Netherlands

2 Department of Internal Medicine – Geriatrics, Amsterdam UMC, location VUmc, Amsterdam, The Netherlands

3 Amsterdam Cardiovascular Sciences, Diabetes & Metabolism, Atherosclerosis & Ischemic Syndromes, Amsterdam, The Netherlands

4 Department of Experimental Vascular Medicine, Amsterdam UMC, location AMC, Amsterdam, The Netherlands

5 Amsterdam Gastroenterology Endocrinology Metabolism, Amsterdam UMC, University of Amsterdam, Amsterdam, Netherlands

6 Amsterdam institute for Infection and Immunity, Infectious diseases, Cancer Immunology, Amsterdam, the Netherlands

7 Department of Cardiology, Angiology and Intensive Care Medicine, Deutsches Herzzentrum der Charité, Campus Benjamin Franklin, Berlin, Germany

8 German Center for Cardiovascular Research (DZHK), Partner Site Berlin, Berlin, Germany

9 Friede Springe-Cardiovascular Prevention Center at Charité, Charité-Universitätsmedizin Berlin Institute of Health (BIH), Berlin, Germany

10 Department of Cardiovascular & Metabolic Sciences, Lerner Research Institute, Cleveland Clinic, Cleveland, Ohio, USA

11 Department of Cardiovascular Medicine, Heart, Vascular and Thoracic Institute, Cleveland Clinic, Cleveland, Ohio, USA

12 Department of Internal medicine, Division of Pharmacology, Erasmus MC, Rotterdam, The Netherlands

13 Department of Public and Occupational Medicine, Amsterdam UMC, Amsterdam, The Netherlands

* **Corresponding author**: Barbara J.H. Verhaar, MD, Department of Vascular Medicine, Amsterdam UMC – Location AMC, PO box 22660, 1100 DD, Amsterdam, The Netherlands, E-mail: b.j.verhaar@amsterdamumc.nl

Supplementary Methods

Participant recruitment

Participants were recruited between March 2021 and July 2022 through online advertising. The study visits took place in the Amsterdam UMC, Amsterdam, the Netherlands. All screened participants provided written informed consent and all study procedures were approved by the institutional review board of the Amsterdam UMC location AMC. The study was registered in the Dutch Trial Register (#NL8924; https://clinicaltrialregister.nl/nl/trial/22936). We included Dutch participants aged 40 to 65 with hypertension (office systolic BP between 140 and 160 mmHg and/or diastolic BP between 90 and 100 mmHg) without treatment, or the use of one BP lowering drug. The BP cut-off aligns with grade I hypertension in the 2018 ESC/ESH hypertension guidelines, while in the 2017 ACC/AHA guidelines, these values would classify as grade II hypertension.[1,2] Participants were required to have a body mass index (BMI) below 27 kg/m^2. Postmenopausal women were eligible for the study, while premenopausal women were not included due to potential hormonal influences on both blood pressure and gut microbiota composition.[3,4] Exclusion criteria were the use of beta blockers, current smoking, known secondary causes of hypertension, a history of cardiovascular disease, impaired renal function, diabetes or severe gastro-intestinal disease. Patients on BP lowering drugs were asked to discontinue this medication for the duration of the study, with a four-week wash-out period monitored by weekly home BP measurements before the baseline visit. Following the Dutch general practice guideline, individuals diagnosed with hypertension and deemed to have low cardiovascular risk were initially advised on lifestyle changes as a first-line treatment prior to the start of antihypertensive medication. Some untreated participants were still in this stage during recruitment. Others had previously used antihypertensive medication but had chosen to discontinue the treatment, often driven by their dissatisfaction with antihypertensive drug therapy.

Power calculation and impact of SARS-CoV-2 pandemic

The primary outcome was defined in the study protocol as daytime 24-hour systolic BP. Prior to the study, we performed a power calculation using G*Power. We calculated that we would need 45 subjects to find a 5-mmHg difference on daytime 24-hour systolic BP after four weeks of treatment with sodium butyrate, with 80% power and alpha 0.05. We aimed for 50 participants to allow for a 10% drop-out rate. Due to the severe acute respiratory syndrome coronavirus 2 (SARS-CoV-2) pandemic, the recruitment of participants progressed slower than expected and the trial had to be terminated prematurely at 23 inclusions due to the end of funding. There was one participant in the placebo group who tested positive for COVID-19 during the study participation, and as a result, the last study visit was a few days delayed. After the end of the lockdown of spring 2021, there have been no further strict lockdowns in the Netherlands and the study was never interrupted. Therefore, we feel that COVID-19 did not bias the study outcomes, and particularly, did not cause imbalance between the groups.

Randomization, blinding and intervention

Included participants were randomized using a stratified alternating block randomization (1:1 ratio) performed in the data collection system Castor EDC. Block sizes were 4, 6, or 8 with two strata for age (≤50 years and >50 years) and sex, to ensure that the two groups had similar baseline risk and were balanced in numbers. The study pharmacist (K.W.) could review the treatment allocation of participants in the data collection system in order to prepare the study

medication; a randomization assistant checked and confirmed the treatment allocation. Physicians and study participants were blinded to treatment allocation, until the statistical analyses were completed in a blinded fashion. Participants in the butyrate treatment arm received 3.9 grams of sodium butyrate daily for four weeks, divided into 13 capsules taken in the morning and evening (total of 26 capsules/day). Placebo arm participants received an equal number of identical placebo capsules containing an equivalent amount of sodium chloride (2.03 grams). Both groups had an additional daily sodium load of 798 mg. Treatment compliance was monitored by asking participants after 2 and 4 weeks how many dosing moments they had missed, and by counting the number of remaining capsules in the returned packaging after study completion. In addition, participants were asked if they experienced any adverse effects. Only adverse effects assessed by the investigator as possibly related to the investigational product were reported.

Study visits

Data and samples were collected around four study visits. We instructed participants not to make any impactful changes to their diet during their study participation. The first visit was at baseline, before the start of the intervention. Prior to the visit, participants were asked to keep a nutritional diary for three days (voedingscentrum.nl/eetmeter), and to collect fecal samples and 24 hours urine. The visit took place in the morning after an overnight fast. Office BP was recorded, and participants were weighed. In supine position, body composition was measured using a multi-frequency body impedance analysis (BIA) device, and a five-minute continuous BP recording was made using a finger photoplethysmography device (Nexfin®, Edwards Lifesciences, Irvine, CA). Next, venous blood samples were collected and kept on ice until further processing. Patients were connected to an ambulatory 24-hour measurement device, that measured until the next morning. After completing the 24-hour BP measurement, they were instructed to take the first capsules. The second, midterm visit took place after two weeks of treatment. This was a shorter visit that included a compliance and adverse events check, office BP measurement, weighing and BIA measurement. The third visit took place at end of the intervention after approximately four weeks. All baseline measurements were repeated, in addition to a compliance and adverse events check. The fourth and last visit took place one week after discontinuation of the treatment to assess if the treatment effects would persist longer than the treatment duration.

Blood pressure measurements

Office BP was measured by a semi-automatic oscillometric device (Microlife WatchBP Home; Microlife AG, Switzerland) following the European Society of Hypertension guidelines.[2] Three BP measurements were taken in seated position after 5 minutes of rest and the average BP was calculated from the second and third measurements. 24 hours ambulatory BP measurements were performed with oscillometric Spacelabs 90217 devices (Spacelabs Healthcare, Issaquah, WA). The devices were programmed to measure four times per hour during daytime (from 7.00 am to 10.00 pm) and two times per hour during nighttime (from 10.00 pm to 7.00 am). A valid 24-hour recording was defined as a recording with >70% of the programmed measurements being successful; all recordings in this study were valid. Participants reported their bedtime and waking time to enable the calculation of average daytime and nighttime BP.

Processing of continuous finger BP measurements

From a Nexfin® device, we exported raw beat-to-beat data of inter-beat intervals (IBI), systolic BP and dP/dt. Analyses to calculate heart rate variability (HRV) and baroreceptor sensitivity (BRS) were performed in Matlab (R2019a; The MathWorks, Inc.). A moving average filter was applied to the beat-to-beat data to exclude measurement artefacts and ectopic beats.[6] We excluded recordings of which more than 20% of beats needed to be removed, or if there was no continuous segment of at least 30 beats without internal calibration. Out of 63 recordings (21 participants, each measured at 3 visits), one recording was excluded. To quantify HRV, the standard deviation of normal-to- normal intervals (SDNN) was calculated. BRS was calculated with a cross-correlation method. Using a sliding window, each successive 10 second interval of IBI was cross-correlated with a 10 second interval of systolic BP measurements, with a time shift varying between 0 and 5 seconds, in which systolic BP preceded IBI. The time delay with maximum correlation was chosen, from which cross-correlation estimation of BRS (xBRS) was calculated by dividing the standard deviation (SD) of the IBI by the SD of the systolic BP for that segment. The xBRS of the complete recording was defined as the geometric mean of all segments with significant positive correlation ($p<0.05$).

Sample collection and biochemistry

Blood was collected in Vacutainer tubes containing heparin, ethylenediaminetetraacetic acid (EDTA) or serum gel tubes. Plasma or serum was isolated from blood samples by centrifugation of these tubes at 1500g for 10 min at 4°C. Sodium, potassium, creatinine, total cholesterol, high density lipoprotein (HDL)-cholesterol and triacylglycerols were measured in plasma with commercially available enzymatic assays (Randox, Antrim, UK; and DiaSys, Holzheim, Germany). These analyses were performed using a Selectra 5 autoanalyzer (Sopachem, Ochten, the Netherlands). Low density lipoprotein (LDL)-cholesterol was calculated using the Friedewald formula. EDTA plasma that was not directly used for measurements was stored at -80°C for later analysis (renin, aldosterone, interleukin-6 (IL-6), interferon gamma (IFNγ) and short chain fatty acids (SCFA) measurements). Fecal samples were collected by the participants the day before the study visit and were stored overnight in a freezer. Frozen samples were transported to the hospital in a provided cooling bag with cooling elements. In the hospital, samples were stored at –80°C until further processing. Urine was collected for 24 hours prior to the study visits (baseline, end of intervention and one week follow-up). The total volume was noted and in a sample of the urine collection, sodium and creatinine levels were measured. Fractional excretion of sodium was calculated from urine and plasma sodium and creatinine.[7]

Short chain fatty acids levels

Fecal levels of SCFA were quantified using high-performance liquid chromatography (HPLC) with ultraviolet detection, following the method described by De Baere *et al.*[8] Dry weights for all samples were measured by freeze-drying homogenized fecal aliquots for 24 hours. The concentrations obtained from HPLC measurements were adjusted for variations in dry weight per sample. For plasma SCFA analysis, gas chromatography – mass spectrometry (GC-MS) was employed. Aliquots of 30 µl supernatant were mixed with 50 µl 2-Butanol/Pyridine (3:2) containing six heavy-labeled internal standards. Carboxylic acids were then derivatized with isobutyl chloroformate. Following derivatization, the sample was mixed with hexane and the hexane layer was removed for GC-MS analysis. Quantitation of acetic acid, butyric acid, isovaleric acid, lactic acid, propionic acid, and succinic acid was carried out using isotope dilution GC-MS/MS in multiple reaction monitoring (MRM) mode. Calibration curves were measured for each analyte to determine the absolute quantity of each SCFA. Thermo TSQ-

Evo triple quadrupole in tandem with the Trace 1310 gas chromatograph (Thermo Fisher Scientific) was employed for analysis. Chromatographic separation was achieved by using an HP-5MS fused-silica capillary column (30 m × 0.250 mm × 0.25 µm; Agilent Technologies, Santa Clara, CA, USA) coated with 5% phenylmethyl siloxane. Each extract (1 µl) was injected in split mode (10:1), and the helium carrier gas flow was set at 1 ml/min. The GC oven temperature program was initiated at 40°C, held for two minutes after injection, and then increased to 50°C at a rate of 3°C/minute.[9]

Serotonin levels

Plasma serotonin concentrations were determined in EDTA plasma samples using an HPLC method. A 225 µl aliquot was spiked with Nitro-Tyrosine (Sigma-Aldrich, Amsterdam, Netherlands) as internal standard, followed by precipitation through the addition of 125 µl with Per-Chloric Acid (10%) (Sigma-Aldrich). All samples were vortexed for 10 seconds, incubated on ice for 15 minutes and then centrifuged for 10 minutes at maximum speed. The supernatant (250 µl) was transferred into an HPLC vial for subsequent analysis. All samples and standards (Serotonin; Sigma Aldrich, Darmstadt, Germany) were stored in a Jasco auto sampler (AS4285) at 10ºC. A 10 µl volume was injected into the HPLC system, and metabolite separation was performed with a Microsphere C18 column (100 mm × 4.6 mm, dp: 3 µm, Agilent Technologies, Amstelveen, Netherlands). The column temperature was maintained at 30 ºC, and variable flow conditions (0.8-1.5 ml/min) were applied for the mobile phase (NaAc,15 mM, pH 5.5)/Acetonitrile mix using a Jasco quaternary pump (PU4285) during each run. The acetonitrile gradient ranged from 1% to 20 %. Detection was performed at 270 nm with a Jasco UV detector (UV4075). Final data interpretation and quantification were carried out using Chrom-Nav chromatography software (v.2.0).

Renin and aldosterone plasma levels

EDTA plasma samples were shipped at -80°C to the Erasmus MC, Rotterdam, the Netherlands for measurement of aldosterone and renin concentrations. Renin concentrations were measured with an immunoradiometric assay (Beckman Coulter, Immunotech, Prague, Czech Republic) with an active site-directed radiolabeled antibody with high specificity for renin (as opposed to prorenin) and a lower detection limit of 2 pg/ml. Plasma aldosterone levels were measured with a solid-phase radioimmunoassay (Demidetec Diagnostics, Kiel, Germany) with a lower detection limit of 12 pg/ml.

Circulating levels of inflammatory mediators

Serum concentrations of IL-6 and IFNγ were determined with the high-sensitivity human ELISA kits (Invitrogen) according to manufacturer procedure. Fecal calprotectin levels were measured using the Stool Extraction Kit plus and EliA™ Calprotectin 2 fluoroenzymeimmunoassay (Thermo Fisher Scientific, Waltham, MA USA) on a Phadia 250 instrument (Phadia™ Laboratory Systems, Uppsala Sweden).

Monocyte isolation from peripheral blood and ex vivo stimulations

Peripheral blood mononuclear cells (PBMC) were isolated from blood collected 10 ml EDTA tubes using a density gradient centrifugation protocol with Leucosep™ (Greiner Bio-One GmbH., Kremsmünster, Austria) tubes filled with 15 ml Lymphoprep™ density gradient medium (Serumwerk Bernburg AG, Bernburg, Germany) under the porous barrier (after centrifugation at 1000g for 30 seconds). The whole blood samples (10 ml) were diluted with

20 ml PBS supplemented with 2 mM EDTA and transferred to the Leucosep™ tubes. after which a layer of PBMCs was formed by centrifuging at 1000g for 15 minutes without brake (4°C). PBMCs were washed twice using PBS with 2 mM EDTA with centrifugation at 350g for 10 minutes (4°C). The cells were frozen in cryovials with heat-inactivated fetal bovine serum (HI-FBS) containing 20% DMSO. The cryovials were placed in a Mr. Frosty container with isopropylalcohol for 24 hours at -80°C and were then moved to the nitrogen storage.

After study completion, PBMC samples were thawed in 10ml of 10% HI-FBS-RPMI 1640 medium. DMSO was removed by centrifugation at 350g for 10min at 4°C. Monocytes were isolated using the Pan Monocyte Isolation Kit and MACS® MS columns (Miltenyi Biotec, Bergisch Gladbach, Germany) according to manufacturer's procedure for the simultaneous isolation of untouched monocyte populations, including classical (CD14+CD16–), non-classical (CD14dimCD16+) and intermediate (CD14+CD16+) monocytes. To determine the count and proportions of monocyte subsets, isolated monocytes were incubated for 10 minutes with Human TruStain FcX Fc Receptor Blocking solution (BioLegend). PBMC were stained in 200 µl 1% BSA in 2 mM EDTA-PBS for 20 minutes at 4°C with the following antibodies: Alexa Fluor® 488 anti-human CD11b Antibody, APC anti-human CD14 Antibody, PerCP/Cyanine5.5 anti-human CD16 Antibody (Biolegend, dilution 1:100). Afterwards, cells were washed three times in in 200 µl 1% BSA in 2 mM EDTA-PBS and fixed for 15 minutes at room temperature in 2% paraformaldehyde in PBS. Stained cells were subsequently analyzed on a BD LSRFortessa™ Cell Analyzer and data analysis was performed with the FlowJo 10.1r5 software (Tree Star). The proportions of classical (CD14+CD16-), intermediate (CD14+CD16+) and nonclassical (CD14dimCD16+) monocytes were calculated as proportions of the total monocyte number.

To assess the monocyte inflammatory profile, monocytes were seeded in 96 well plates and stimulated with either control medium (200 µl of 5% HI-FBS-RPMI 1640 medium with GlutaMAX and Penicillin-Streptomycin), 100 µM palmitate or 1 ng/ml lipopolysaccharide (LPS) for 24 hours in a 37 °C 5% CO_2 incubator. The supernatant was harvested and stored at -80°C; adherent monocytes where washed and the medium replaced. After five days, the monocytes were restimulated with LPS 10 ng/ml for 24 hours, to induce trained immunity in the palmitate-trained monocytes and immunotolerance in the LPS-trained monocytes. Concentrations of IL-6 and tumor necrosis factor alpha (TNFα) were measured in the collected monocyte supernatant using the OptEIA™ Human TNFα and IL-6 ELISA sets (BD Biosciences), according to the manufacturer instructions. All stimulations were performed in quadruplicate and the average concentrations of IL6 and TNF-alpha per condition were normalized for monocyte count. Cytokine concentrations from the first stimulation are shown as the difference between stimulated and control conditions, and the data from the second stimulation (following resting period) are shown as fold-changes compared to the first stimulation.

T cell phenotyping by flow cytometry

Changes in T cell subsets upon placebo or butyrate treatment were investigated using a 12-color panel designed to identify all major CD4 T populations. One vial of frozen PBMC was thawed and one million PBMCs were used for the staining. PBMCs were fist stained with a cocktail of fluorochrome-labelled antibodies (BV421-conjugated anti-CD194/CCR4, BB700-conjugated anti-CD185/CXCR5, BV786-conjugated anti-CD196/CCR6, PE-conjugated anti-CD294/CRTH2, PE-Cy7-conjugated anti-CD183/CXCR3 (BDBiosciences)) for 10 minutes at 37C in the dark; afterwards a new cocktail of fluorochrome-labelled antibodies (BV605-conjugated anti-CD45RA, BV711-conjugated anti-CD127, BB515-conjugated anti-CD25,

APC-conjugated anti-CCR10, Alexa Fluor 700-conjugated anti-CD3, APC-H7-conjugated anti-CD4, PE-Cy5-conjugated anti-CD8 (BDBiosciences)) was added to pre-stained cells for additional 20 minutes at room temperature in the dark. Stained PBMCs were analyzed using a 4-laser FACSymphony™ A1 Cell Analyzer. The obtained flow cytometric data were analyzed using the FlowJo v10 software. Gating strategies can be found in **Figure S13**.

Gut microbiota composition

DNA was isolated from 150 mg aliquots of fecal samples with a repeated bead-beating protocol, followed by purification with a Maxwell RSC Whole Blood DNA kit.[10] A single-step PCR protocol targeted the V3-V4 region to generate 16S rRNA gene amplicons.[11] The resulting PCR products were purified using Ampure XP beads and then equimolarly pooled. Subsequently, libraries were sequenced on the Illumina MiSeq platform with V3 chemistry and 2x251 cycles. Sequences were processed using a VSEARCH (v.2.15.2) pipeline, merging paired-end reads with a maximum difference set to 100 and allowing for staggered overlap.[12] Merged reads with less than 1.5 expected errors per read were clustered per sample using the cluster_unoise algorithm with a minimum size of 4. Chimeras were removed using the UCHIME3 de-novo method. Amplicon sequence variants (ASV) abundances were determined by mapping all data against the collectively inferred ASV sequence set using the usearch_global algorithm with a 0.97 distance cut-off. Taxonomy was assigned using R (v.4.0.5) and the DADA2 assignTaxonomy function utilizing the SILVA reference database (v.132). Multiple sequence alignment was performed using MAFFT (v.7.511), and the phylogenetic tree was constructed using iqtree (v.2.2.0). The ASV table, taxonomy assignments and phylogenetic tree were integrated into a phyloseq object. After rarefaction to 14,000 counts per sample, this dataset was composed of 62 samples and 2405 ASVs.

Statistics

All statistical analyses were performed in RStudio (v.2023.9.1.494) using R (v.4.2.1). All scripts were made publicly available in a Github repository (https://github.com/barbarahelena/beam-study). Baseline data were presented as mean±SD for continuous variables with normal distribution, median [interquartile range] for continuous variables with non-normal distributions or n (%) for categorical variables, which were calculated with the tableone package (v.0.13.2). We did not use statistical test to assess whether group differences at baseline were likely to occur due to chance, since the groups were randomized and differences could therefore only have been caused by chance. Treatment effects of oral butyrate on all primary and secondary outcomes were assessed using linear mixed models (lme4 (v.1.1.31) and afex (v.1.2.0) packages). All graphs were drawn using ggplot2 (v.3.4.0), ggsci (v.2.9), and ggpubr (v.0.5.0) packages. Missing data in these analyses were limited to one missing continuous BP recording at the baseline time point. This subject was therefore excluded from the xBRS and HRV analyses. Renin levels and ARR were log10-transformed because of their nonnormal distributions. In the unadjusted linear mixed models, participant number was included as fixed effect and the treatment group, time and the interaction term group*time were included as random effects. The effect of treatment was considered significant if the group*time estimate was significant ($p<0.05$). In the adjusted model for ambulatory and office BP, the covariates age, sex, BMI, sodium intake (nutritional diary) and compliance (number of capsules left) were included. The group*time estimates of these models were visualized in a forest plot with 95% confidence intervals (95%-CI). In the model for plasma SCFA levels, we included compliance and the time of the study visit (as proxy for time from morning dose to sample collection) as covariates. In the model for fecal SCFA levels, we only included compliance as covariate, since samples were

already collected before the study visit. For gut microbiota composition, we first calculated beta diversity using weighted UniFrac and tested differences between visits using PERMANOVA. Several alpha diversity indices were calculated using the vegan package (v.2.6.4), including Shannon index and Faith's phylogenetic diversity. Differences between groups at the different time points were tested with Mann-Whitney-U tests. After calculating relative abundances (% of total counts), filtering for a minimal relative abundance of 0.05% in 30% of participants, and log2-transformation, we used linear mixed models to test differences in 132 ASVs over time (group*time interaction). We performed a Benjamini-Hochberg multiple testing correction on the linear mixed model p-values ($q<0.05$).

Data availability

The raw 16S sequencing data are available in the European Nucleotide Archive (ENA), accession number PRJEB60224.

References

1. 2017 ACC/AHA/AAPA/ABC/ACPM/AGS/APhA/ASH/ASPC/NMA/PCNA Guideline for the Prevention, Detection, Evaluation, and Management of High Blood Pressure in Adults: A Report of the American College of Cardiology/American Heart Association Task Force on Clinical Practice Guidelines. *Hypertension*. Published online 2017. Accessed February 6, 2024. https://www.ahajournals.org/doi/full/10.1161/HYP.0000000000000065

2. The Task Force for the management of arterial hypertension of the European Society of Cardiology (ESC) and the European Society of Hypertension (ESH). 2018 ESC/ESH Guidelines for the management of arterial hypertension. *European Heart Journal*. 2018;39(33):3021-3104. doi:10.1093/eurheartj/ehy339

3. Valeri F, Endres K. How biological sex of the host shapes its gut microbiota. *Frontiers in Neuroendocrinology*. 2021;61:100912. doi:10.1016/j.yfrne.2021.100912

4. Prabhushankar R, Krueger C, Manrique C. Membrane Estrogen Receptors: Their Role in Blood Pressure Regulation and Cardiovascular Disease. *Curr Hypertens Rep*. 2013;16(1):408. doi:10.1007/s11906-013-0408-6

5. Faul F, Erdfelder E, Lang AG, Buchner A. G*Power 3: A flexible statistical power analysis program for the social, behavioral, and biomedical sciences. *Behavior Research Methods*. 2007;39(2):175-191. doi:10.3758/BF03193146

6. Collard D, Westerhof BE, Karemaker JM, et al. Automated analysis of finger blood pressure recordings provides insight in determinants of baroreflex sensitivity and heart rate variability—the HELIUS study. *Med Biol Eng Comput*. Published online January 23, 2023. doi:10.1007/s11517-023-02768-4

7. Schreuder MF, Bökenkamp A, Wijk JAE van. Interpretation of the Fractional Excretion of Sodium in the Absence of Acute Kidney Injury: A Cross-Sectional Study. *NEF*. 2017;136(3):221-225. doi:10.1159/000468547

8. De Baere S, Eeckhaut V, Steppe M, et al. Development of a HPLC-UV method for the quantitative determination of four short-chain fatty acids and lactic acid produced by intestinal bacteria during in vitro fermentation. *Journal of Pharmaceutical and Biomedical Analysis*. Published online 2013. doi:10.1016/j.jpba.2013.02.032

9. Lieber AD, Beier UH, Xiao H, et al. Loss of HDAC6 alters gut microbiota and worsens obesity. *The FASEB journal*. 2019;33(1):1098. doi:doi:10.1096/fj.201701586R

10. Costea PI, Zeller G, Sunagawa S, et al. Towards standards for human fecal sample processing in metagenomic studies. *Nature biotechnology*. 2017;35(11):1069-1076.

11. Kozich JJ, Westcott SL, Baxter NT, Highlander SK, Schloss PD. Development of a dual-index sequencing strategy and curation pipeline for analyzing amplicon sequence data on the MiSeq Illumina sequencing platform. *Applied and environmental microbiology*. 2013;79(17):5112-5120.

12. Bokulich NA, Kaehler BD, Rideout JR, et al. Optimizing taxonomic classification of marker-gene amplicon sequences with QIIME 2's q2-feature-classifier plugin. *Microbiome*. 2018;6(1):90. doi:10.1186/s40168-018-0470-z

Supplementary figures

Figure S1: Study recruitment flowchart

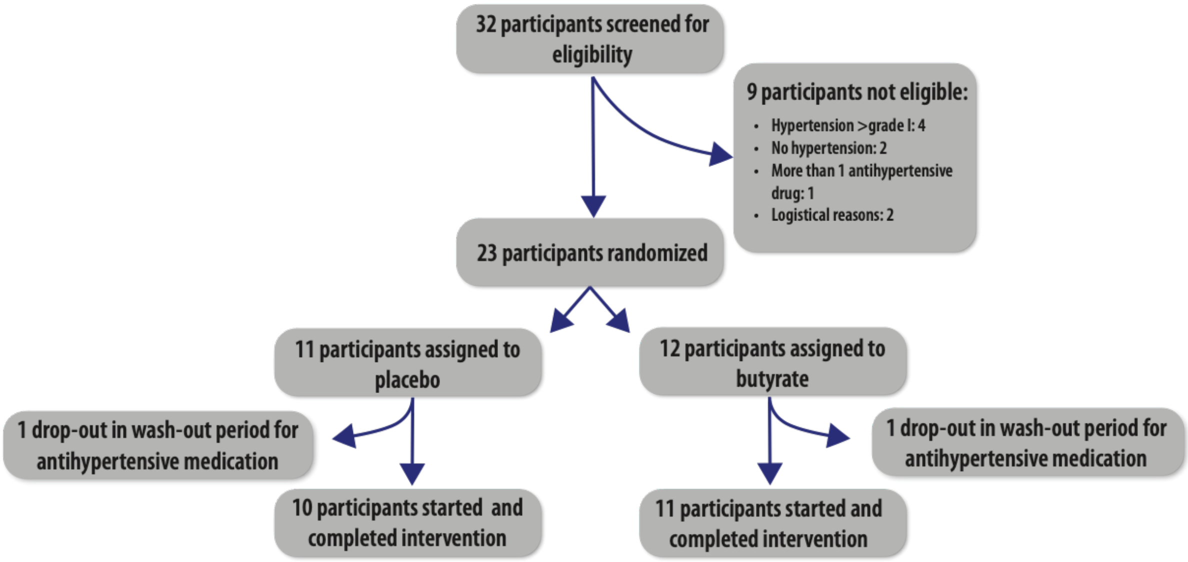

Figure S2: Dietary data

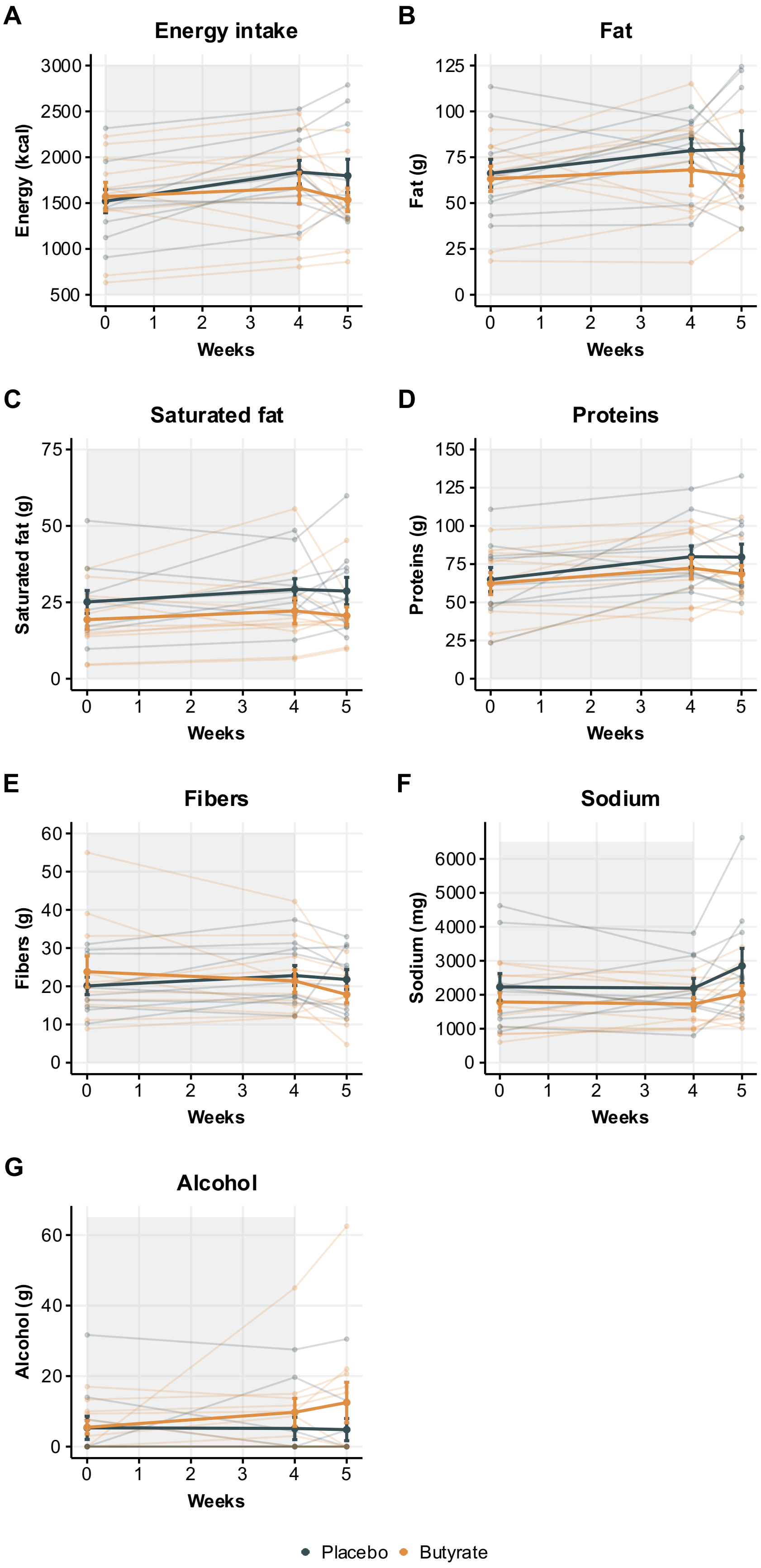

*Daily dietary intake at baseline, at the end of the intervention (week 4) and after the intervention (week 5). Participants filled in the online nutritional diary for three consecutive days before the study visits; the daily intake was averaged over three days. Grey marked area is the time of intervention (placebo or butyrate). The darker lines represent the group means over time, while the lighter lines indicate the individual subjects' changes over time. Error bars represent standard errors (±1 SE) of the means. Differences between groups over time (group*time) were tested with an unadjusted linear mixed model (none of the models were significant).*

Figure S3: Adjusted linear mixed models

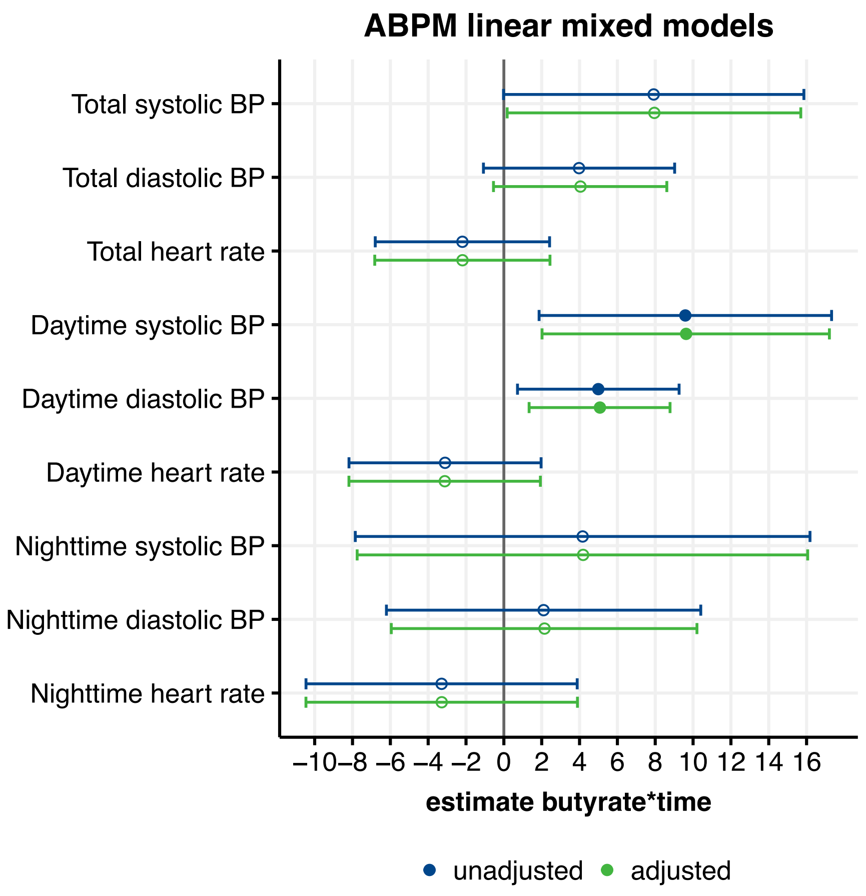

*Estimates with 95% CI (for blood pressure mmHg; for heart rate beats per minute) of linear mixed models (group*time, baseline to end of intervention). The adjusted linear regression model included age, sex, BMI, sodium intake and compliance as covariates.*

Figure S4: Office BP

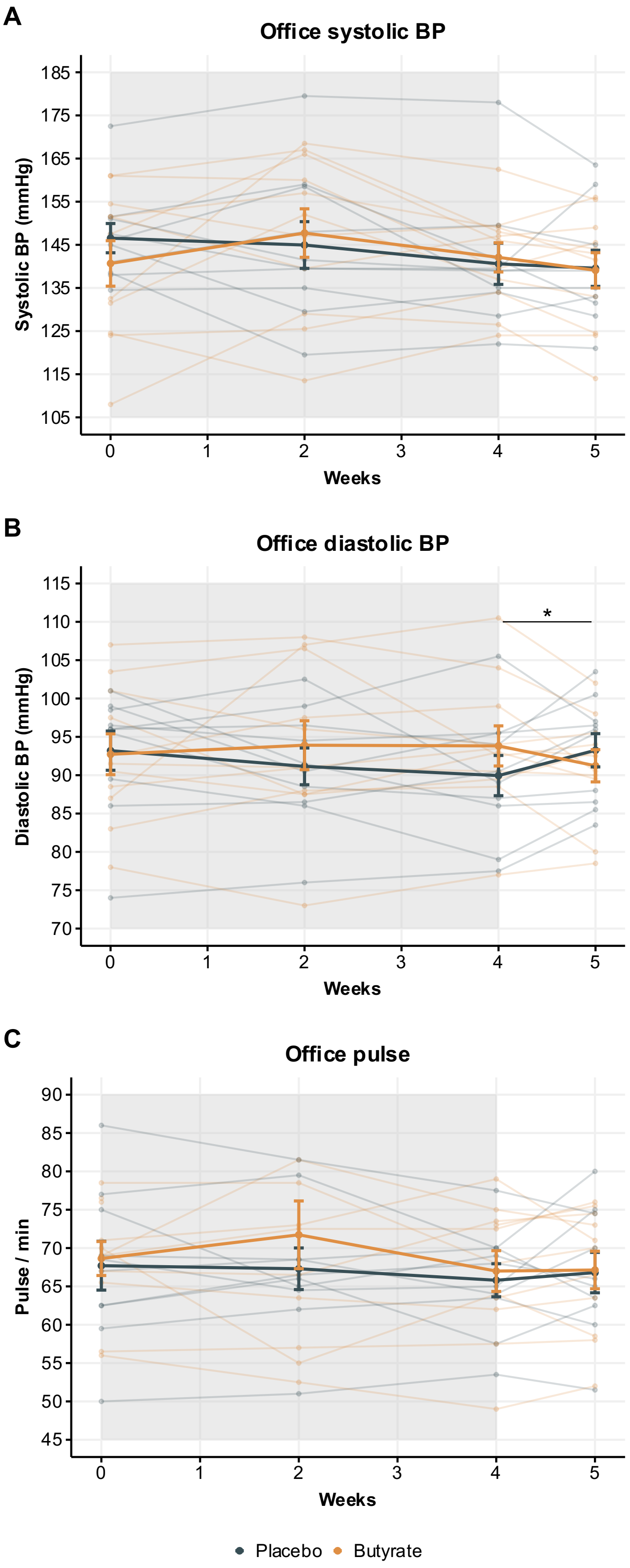

*Office blood pressure (BP) at baseline (week 0), at midterm (week 2), at the end of the intervention (week 4) and after the intervention (week 5). Office BP was measured three times and was averaged over the last two measurements. Grey marked area is the time of intervention (placebo or butyrate). The darker lines represent the group means over time, while the lighter lines indicate the individual subject's changes over time. Error bars represent standard errors (±1 SE) of the means. Differences between groups over time (group*time) were tested with an unadjusted linear mixed model. * = p-value<0.05.*

Figure S5: Body impedance analysis

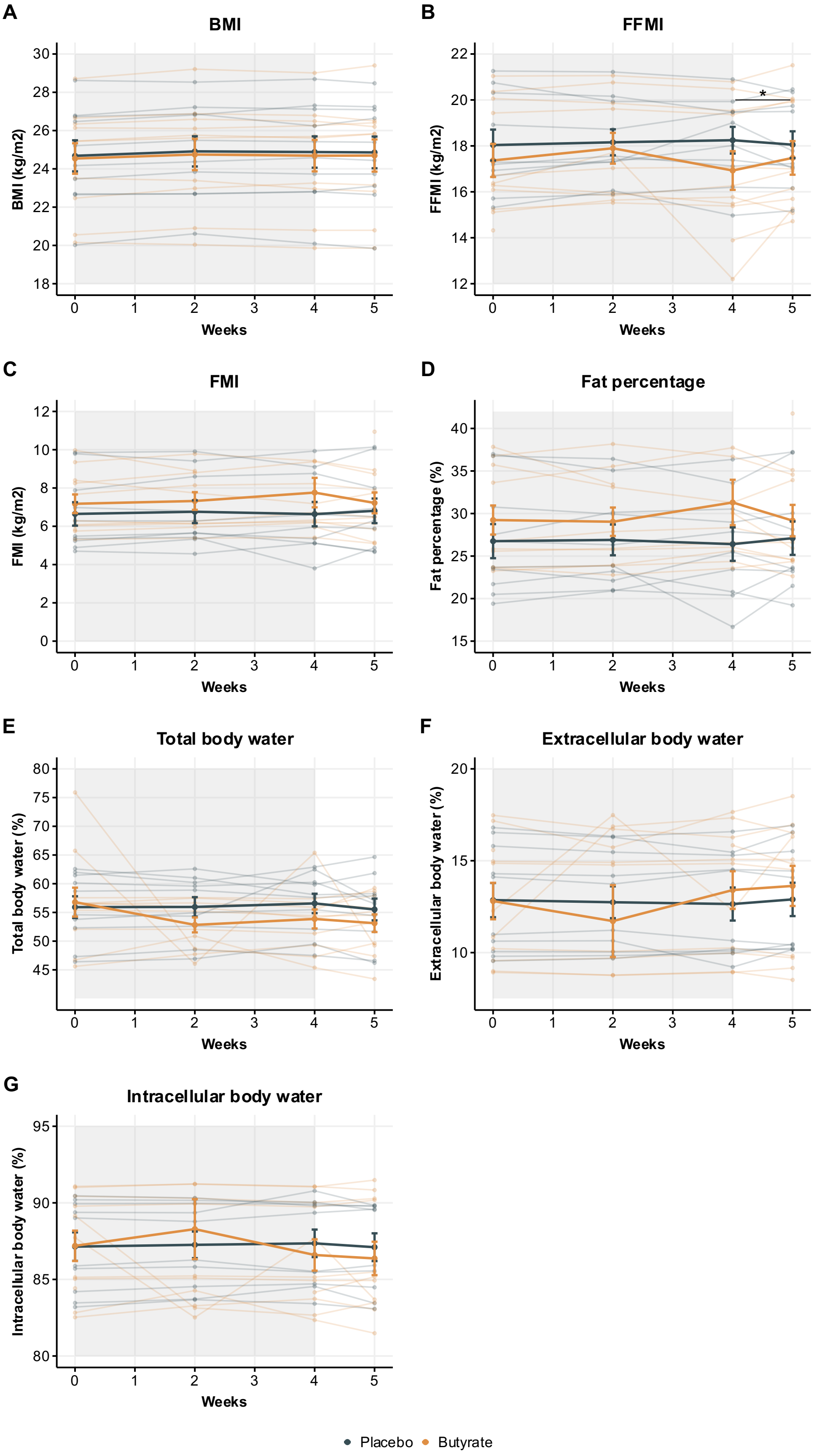

*Parameters calculated from multi-frequency body impedance analysis (BIA) at baseline, at midterm (week 2), at the end of the intervention (week 4) and after the intervention (week 5). The grey marked area is the time of intervention (placebo or butyrate). The darker lines represent the group mean over time, with error bars for standard errors, while the lighter lines indicate the individual subject's changes over time. Differences between groups over time (group*time) were tested with a linear mixed model. * = p-value <0.05. BMI = body mass index, FFMI = fat-free mass index, FMI = fat mass index.*

Figure S6: Serotonin plasma levels

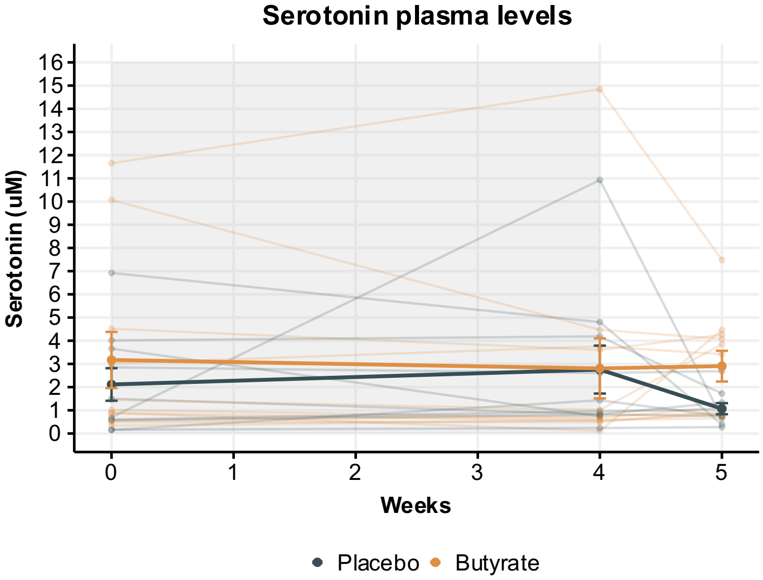

*Plasma serotonin levels at baseline, at midterm (week 2), at the end of the intervention (week 4) and after the intervention (week 5). The grey marked area is the time of intervention (placebo or butyrate). The darker lines represent the group mean over time, with error bars for standard errors, while the lighter lines indicate the individual subject's changes over time. Differences between groups over time (group*time) were tested with linear mixed models (not significant).*

Figure S7: Changes in gut microbiota composition

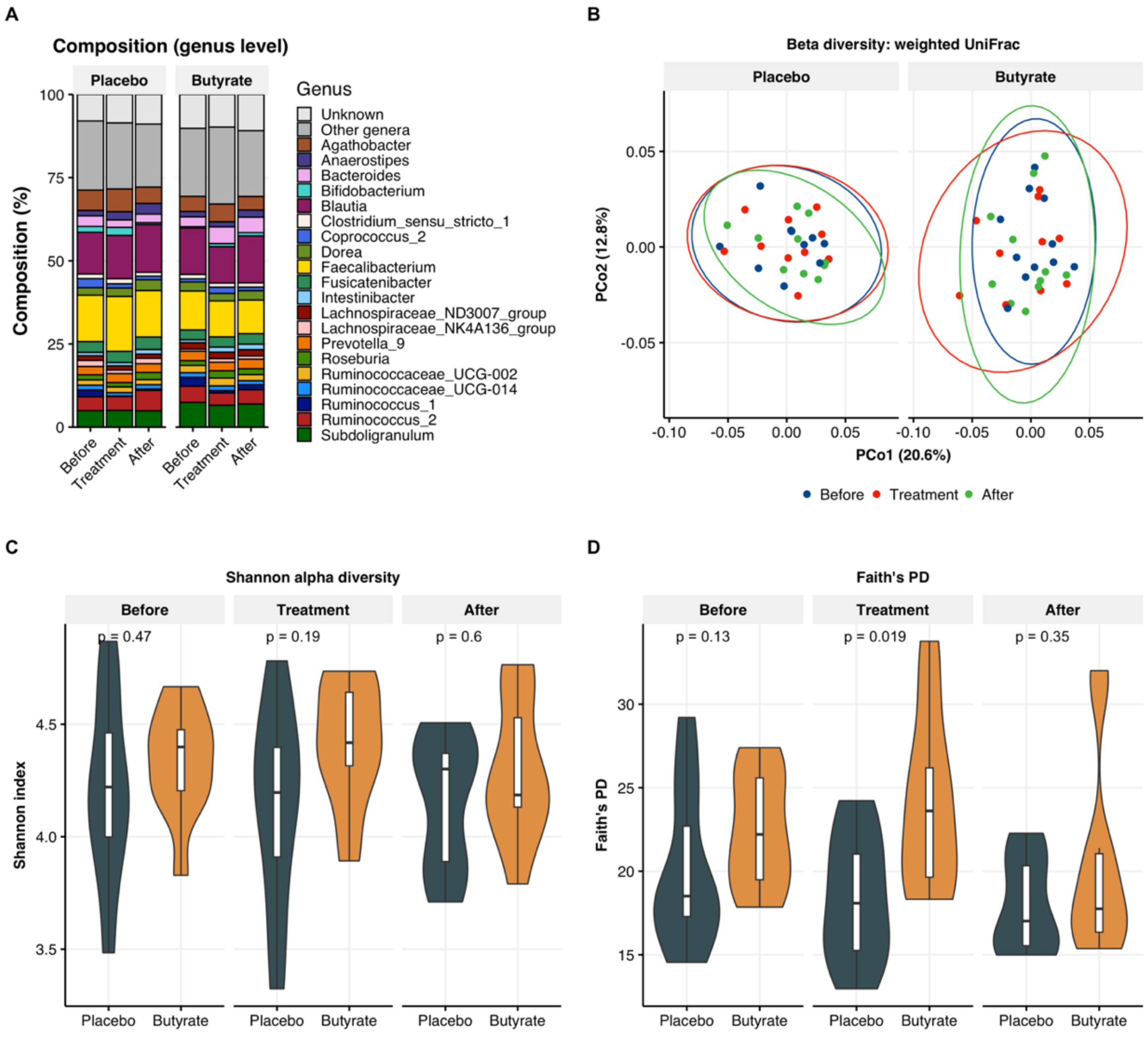

Changes in gut microbiota composition, beta diversity and alpha diversity. A. The gut microbiota composition as determined with 16S rRNA sequencing at different time points (before, during treatment, 1 week after intervention). B. Weighted UniFrac beta diversity at the same time points plotted per group; different colors indicate the three time points. There were no group differences or differences between time points. C. Shannon index as measure of alpha diversity compared between the groups at different time points, tested with Mann-Whitney U tests. D. Faith's phylogenetic diversity compared between the groups at different time points, tested with Mann-Whitney U tests.

Figure S8: Gut microbiota abundance

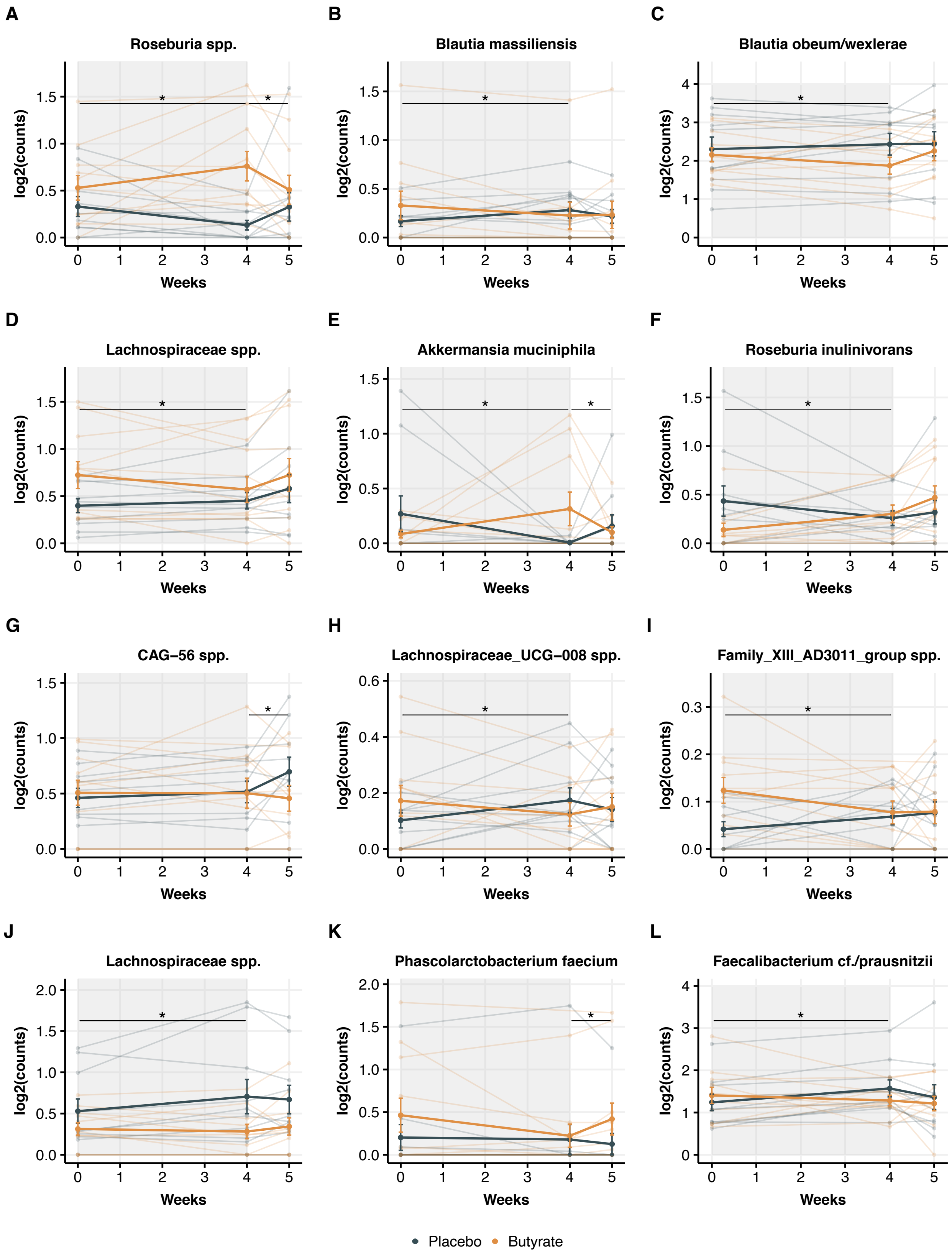

Abundance of microbiota at baseline (week 0), at the end of the intervention (week 4) and after the intervention (week 5). The relative abundance is shown as log2-transformed counts (after adding 1 pseudocount). These were the amplicon sequence variants (ASVs) that were

*significantly different before false discovery rate (FDR) correction. Grey marked area is the time of intervention (placebo or butyrate). The darker lines represent the group means over time, while the lighter lines indicate the individual subjects' changes over time. Error bars represent standard errors (±1 SE) of the means. Differences between groups over time (group*time) were tested with an unadjusted linear mixed model. * = p-value<0.05 without FDR correction.*

Figure S9: Proportions of monocyte subpopulations

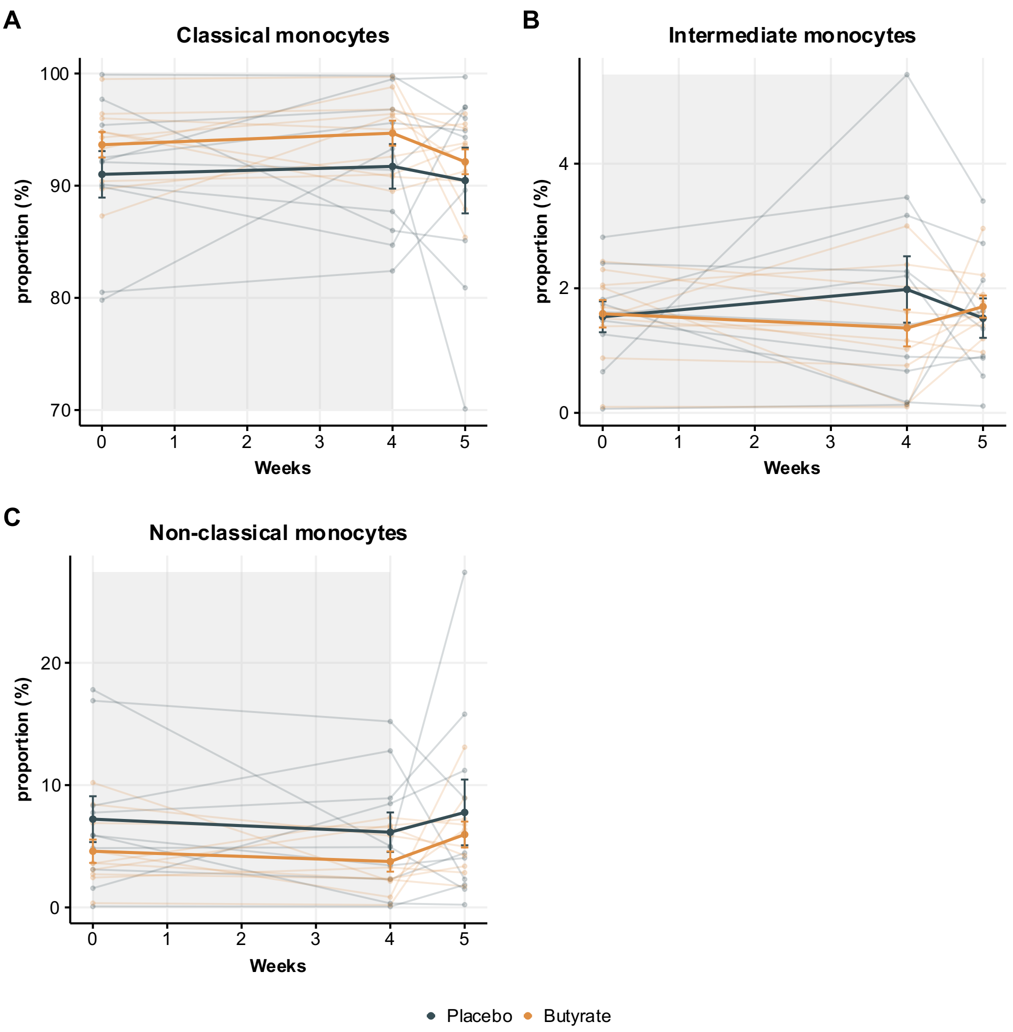

*Proportions of subpopulations of monocytes at baseline (week 0), at the end of the intervention (week 4) and after the intervention (week 5). Classical subpopulation of monocytes is CD14+CD16-, intermediate CD14+CD16+ and nonclassical CD14-CD16+. Grey marked area is the time of intervention (placebo or butyrate). The darker lines represent the group means over time, while the lighter lines indicate the individual subjects' changes over time. Error bars represent standard errors (±1 SE) of the means. Differences between groups over time (group*time) were tested with an unadjusted linear mixed model (none of the models were significant).*

Figure S10: Serum interleukin-6 levels

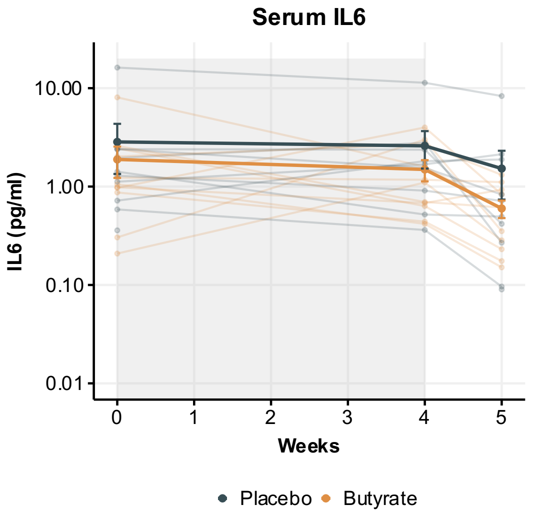

*Levels of serum interleukin-6 (IL6) at baseline, at the end of the intervention (week 4) and after the intervention (week 5). The y-axis is log-scaled with the original measurement unit shown (pg/ml). The grey marked area is the time of intervention (placebo or butyrate). The darker lines represent the group means over time, while the lighter lines indicate the individual subjects' changes over time. Error bars represent standard errors (±1 SE) of the means. Differences between groups over time (group*time) were tested with a linear mixed model (not significant).*

Figure S11: T cell immune profile

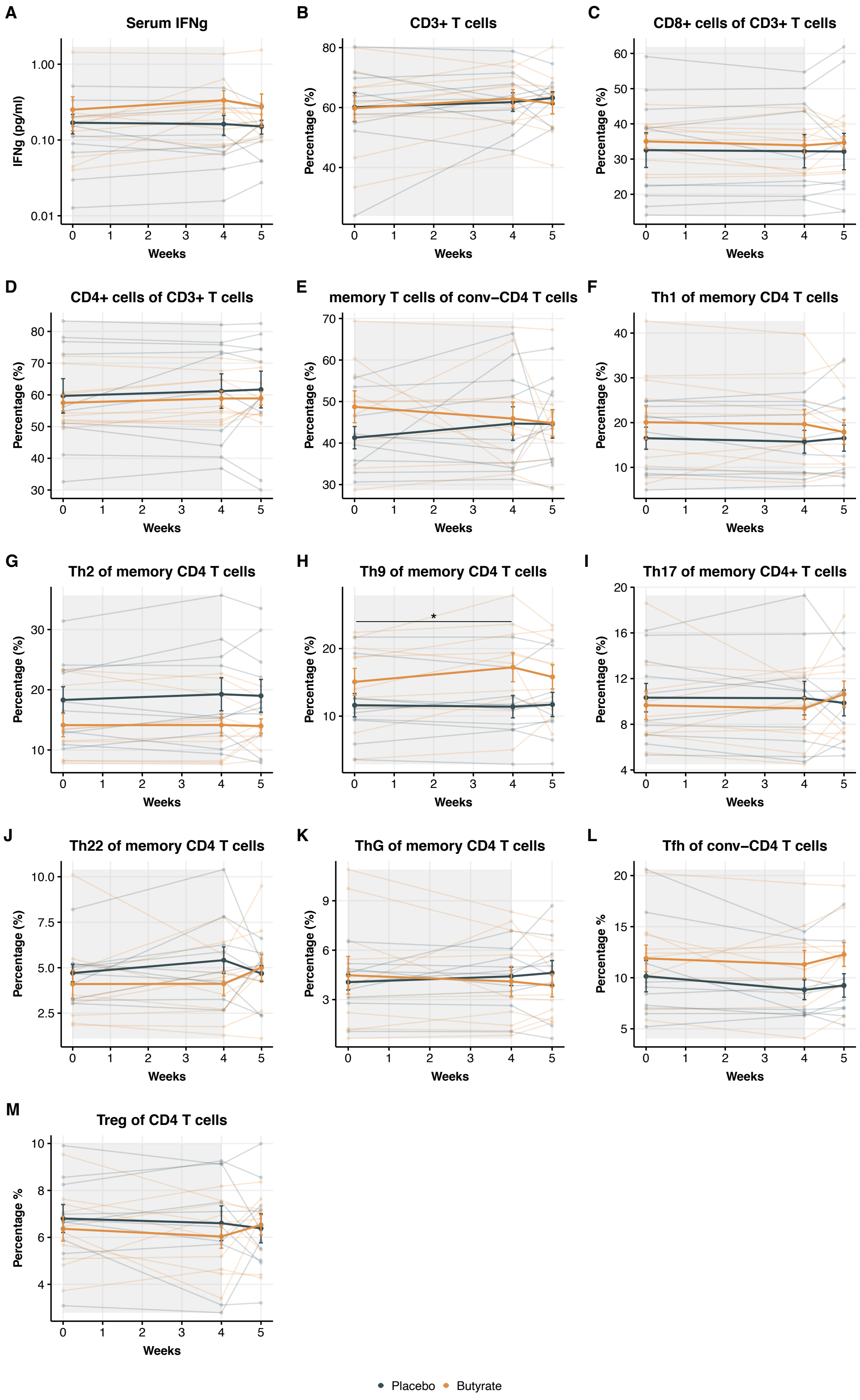

*T cell immune profile parameters at baseline, at the end of the intervention (week 4) and after the intervention (week 5). The y-axis of interferon gamma (IFNg) is log10-scaled with the original measurement unit (pg/ml). The grey marked area is the time of intervention (placebo or butyrate). The darker lines represent the group means over time, while the lighter lines indicate the individual subjects' changes over time. Error bars represent standard errors (±1 SE) of the means. Differences between groups over time (group*time) were tested with a linear mixed model (only the model for Th9 cells was significant between 0 and 4 weeks). * = p-value<0.05*

Figure S12: Specific subsets of T cells

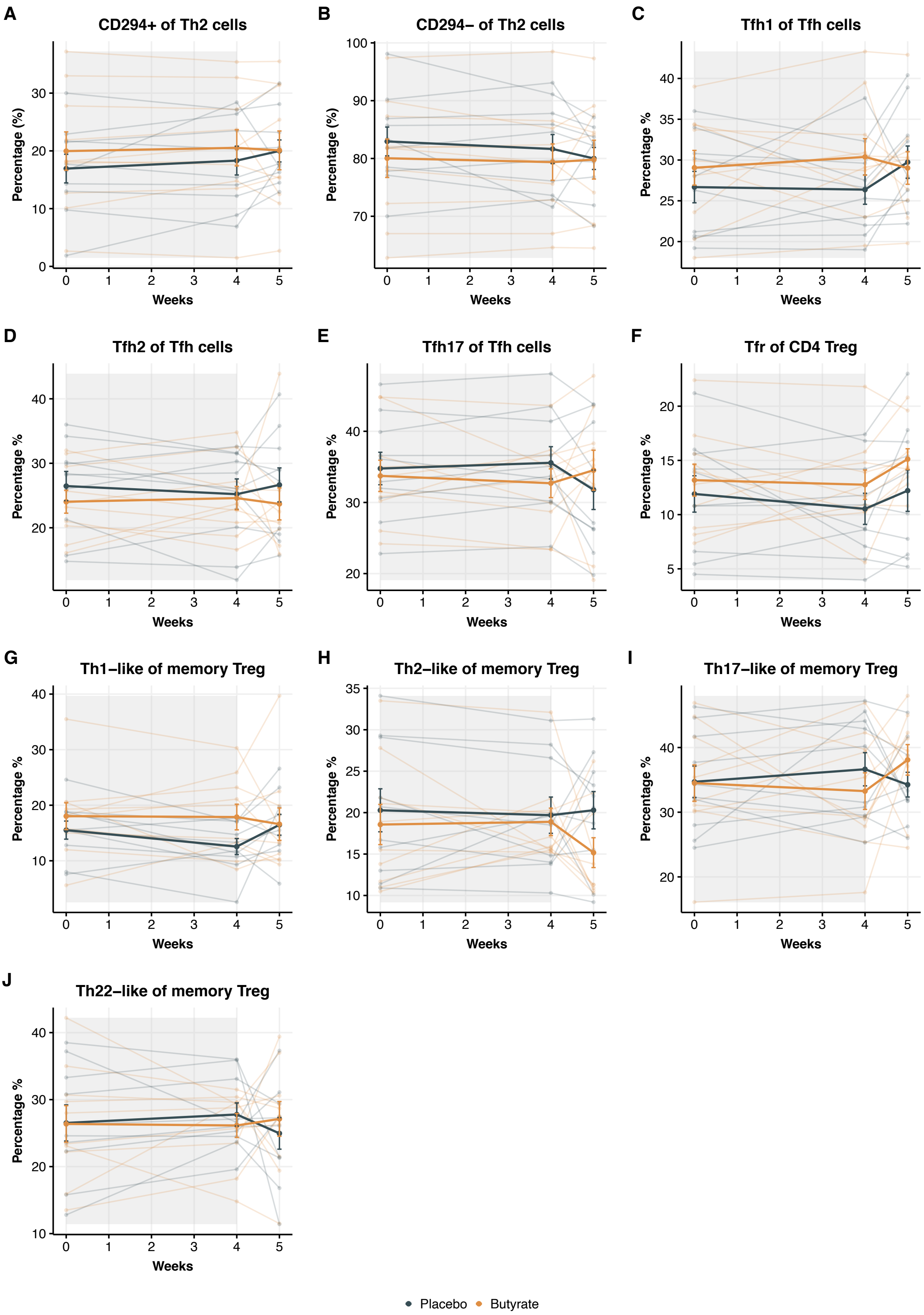

*Proportions of specific subsets of T cells at baseline, at the end of the intervention (week 4) and after the intervention (week 5). The grey marked area is the time of intervention (placebo or butyrate). The darker lines represent the group means over time, while the lighter lines indicate the individual subjects' changes over time. Error bars represent standard errors (±1 SE) of the means. Differences between groups over time (group*time) were tested with a linear mixed model (none of the models were significant).*

Figure S13: Calprotectin levels

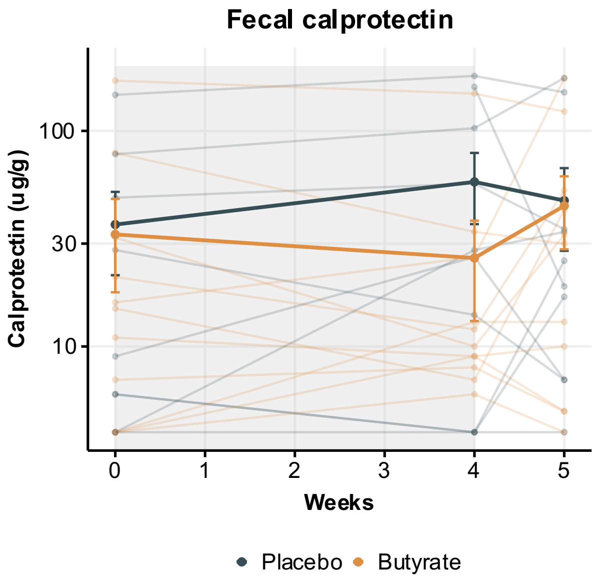

*Levels of fecal calprotectin at baseline, at the end of the intervention (week 4) and after the intervention (week 5). The y-axis is log10-scaled with the original measurement unit shown (µg/g). The grey marked area is the time of intervention (placebo or butyrate). The darker lines represent the group means over time, while the lighter lines indicate the individual subjects' changes over time. Error bars represent standard errors (±1 SE) of the means. Differences between groups over time (group*time) were tested with a linear mixed model.*

Figure S14: Gating strategy

A. Monocytes

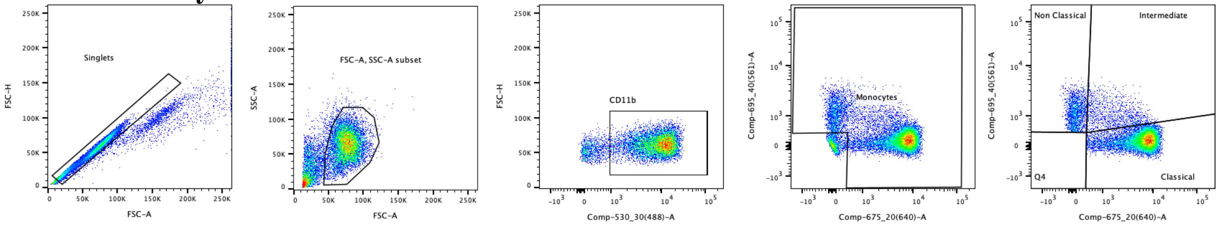

B. T cells

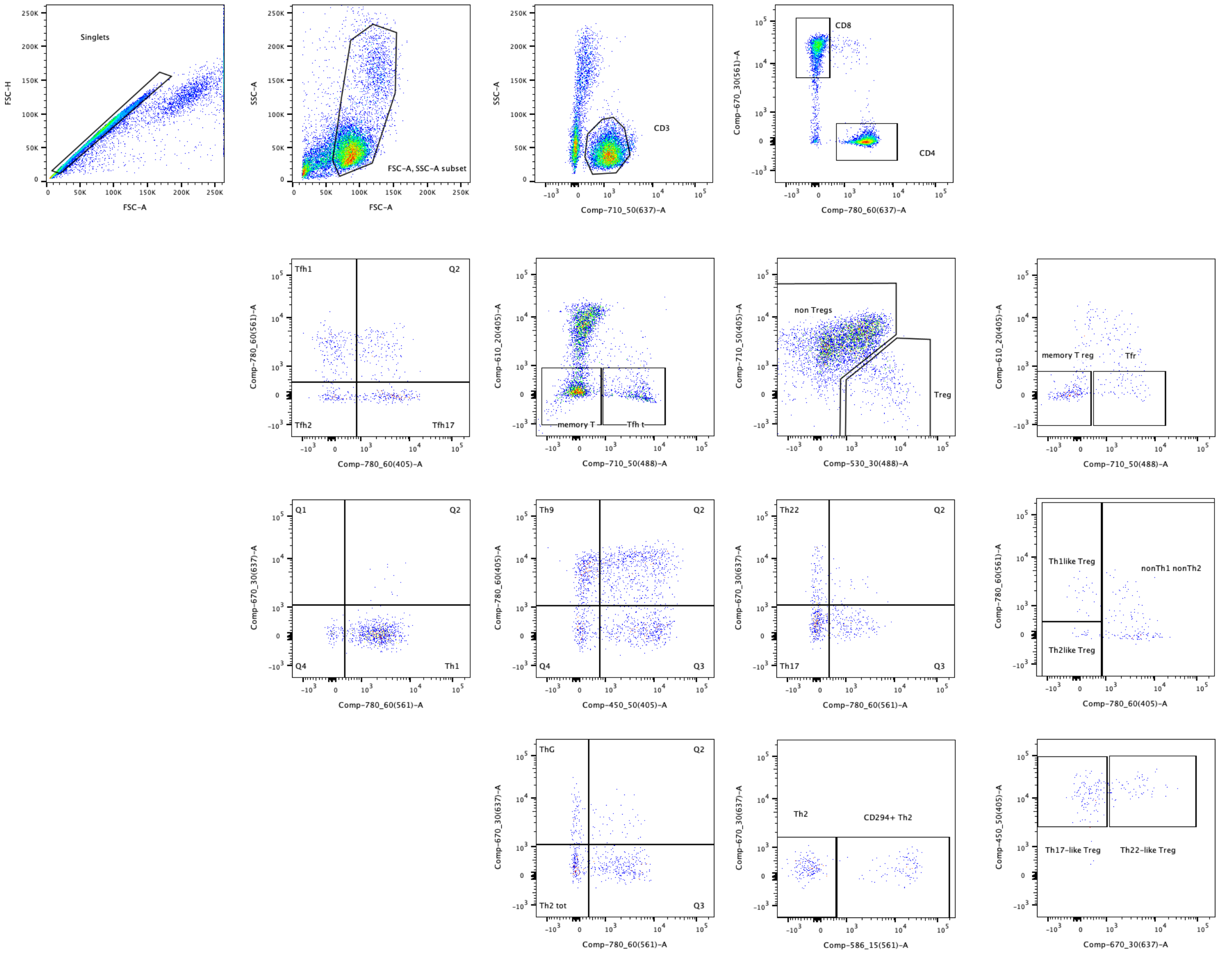

Gating strategies used for flow cytometric analysis of (A) monocytes and (B) T cells.
